# What do people know and think about medical overuse? an online questionnaire study in Germany

**DOI:** 10.1371/journal.pone.0299907

**Published:** 2024-03-07

**Authors:** Carolin Nürnberger, Thomas Kühlein, Susann Hueber

**Affiliations:** 1 Faculty of Medicine, Friedrich-Alexander-University Erlangen Nürnberg (FAU), Erlangen, Germany; 2 Institute of General Practice, Universitätsklinikum Erlangen, Erlangen, Germany; Ekiti State University College of Medicine, NIGERIA

## Abstract

**Background:**

Medical overuse is defined as health care services that exceed the individual needs of patients and when the potential harms of medical interventions exceed their benefits. It has impacts on patients as well as on health care resources. To address medical overuse, it is important to understand the knowledge and experiences of overuse on the side of patients.

**Research questions:**

What is the citizens’ understanding of overuse? How do they assess its relevance, causes, consequences and potential solutions?

**Methods:**

A quantitative online survey was conducted. The participants were asked to state what they understand by medical overuse. Statements on causes, consequences and possible solutions were evaluated. Recruitment was carried out via a panel of a market research institute (Schlesinger Group).

**Results:**

The survey was completed by 406 participants. In terms of age and gender, the sample corresponded to the distribution in the German population. The majority had never heard of medical overuse (58%). About 60% assumed that medical overuse means "too much medicine including overtreatment and overtesting”. Medical overuse was mainly suspected for services not covered by the public health insurance system (56%), surgical interventions (45%) and medication prescriptions (37%). Reasons for medical overuse were seen in uncoordinated care and financial incentives, but also in the expectations of patients. The main problem with medical overuse was seen in rising health care costs, while harmful physical and mental consequences for patients were mentioned less often. In order to reduce medical overuse, little importance was attributed to a primary care based system or higher financial contribution of patients. Instead, stricter cost control on the side of physicians and better coordination between care providers were suggested as solutions. Differences in socio-demographic characteristics hardly showed any differences in response behavior.

**Conclusion:**

More than half of the respondents had never heard of medical overuse. Overuse was mainly associated with financial causes and consequences. It was not seen that overuse can be harmful for patients directly. The limited awareness of the problem of overuse probably is a barrier to tackling it effectively. Communicating the topic to the public might therefore be an effective start to mitigate medical overuse.

**Take home message:**

Many citizens seem not to be familiar with the concept of medical overuse, especially not with the fact that it may directly cause harm to patients. Informing citizens about the harms of medical overuse might be helpful in mitigating it.

## Introduction

### Background

Medical overuse is defined as health care services that exceed the individual needs of patients and when the potential harms of medical interventions exceed their benefits [1]. Despite this seemingly clear definition, measuring overuse bears some difficulties. Some treatments that can cause too much harm to a majority of patients, might be a reasonable option for others or vice versa [2,3]. Reasons for medical overuse can be patient or provider driven. There is a widespread belief among the public, that more care would be better [4]. Country specific health care regulations might mitigate or contribute to overuse. Patients exposed to overuse can experience several types of harm, including physical, psychological, social or financial consequences [5,6]. Overuse accounts for between 10 and 30 percent of all treatments [4], which has a strong impact on overall healthcare resources, both financial and human [6].

To mitigate the consequences of unnecessary medical interventions, the concept of quaternary prevention has been developed [7]. Different guidelines have been established to help physicians decide about appropriate care. A prominent example in Germany is the guideline of the German College of General Practitioners and Family Physicians (DEGAM) “Protection against overuse and underuse of health care–choosing together”, which provides guidance for treatment in primary care [8]. Some guidelines also exist in easy language for patients. There is a number of campaigns that address overuse and comprise related guidelines, such as "Choosing Wisely" by the American Board of Internal Medicine [9], "Too much medicine" by the British Medical Journal [10], and the initiative "Klug entscheiden" by the German Society of Internal Medicine [11].

As suggested by Morgan et al. (2015) research on medical overuse should follow a structured agenda. A crucial step is to explore how medical overuse is perceived in society and to understand where patients and the public see reasons for it and potential for improvement [4]. Surveys among citizens have already been conducted in Australia [12] and the United Kingdom [13]. However, those studies mainly focused on patients’ perceptions of overdiagnosis but not overtreatment and overuse. The term overdiagnosis describes a diagnosis that never would have caused symptoms or harms during a patients’ lifetime. Overdiagnosis triggers overuse in the context of unnecessary further diagnostic and therapeutic measures [14,15]. Both studies revealed that patients have a different understanding of overdiagnosis as compared to the scientific community as well as a low level of understanding about the underlying problem [12,13]. A study among German primary care physicians showed that some do recognize overuse as a problem while those who tend to overuse see themselves being criticized by the term itself and react defensively [16]. Physicians also see little opportunity for counteraction on their own. Instead, they attribute reasons to factors within the healthcare system and to patients’ expectations [17,18]. Patients seem not to be aware of medical overuse and hence do not realize the problematic nature of the issue. They stated that they did not personally experience overuse yet and seem more likely to fear medical underuse than overuse. Many perceived overuse as particularly good care [16,19]. So far, the perception of medical overuse has been investigated qualitatively but larger surveys of the public opinion on medical overuse are still missing.

### Objectives

The study aimed to assess the perception of medical overuse, the understanding of reasons and consequences and to evaluate solutions in the general population in Germany. Furthermore, differences in the opinion on medical overuse due to sociodemographic characteristics and participants’ healthcare behavior were investigated.

## Material and methods

### Study design and setting

An online questionnaire study was conducted. Reporting is based on the STROBE [20] statement for cross-sectional studies and the CHERRIES checklist for Internet E-Surveys [21]; available as (S1 and S2 Files). The study was conducted as a cross-sectional study at the Institute of General Practice at Universitätsklinikum Erlangen, Germany.

A pre-test was performed with seven citizens to evaluate the content, design and functionality of the questionnaire. Participants needed to be aged 18 years or older. Participation was voluntary. The participants of the pre-test were recruited via personal contact. CN and SH discussed all responses and decided whether modifications in the questionnaire would be necessary. Only changes in wordings were made. No question was deleted.

Participants of the study were recruited via collaboration with Schlesinger Group [22], an institute for market research. For sample size calculation G-Power was used [23]. We assumed that differences in outcomes according to gender and to knowledge of medical overuse prior to the study in the perception of overuse will probably be small (Cohen’s *d* = 0.2 to 0.3). To obtain a type 1 error alpha of maximum 5% and a power of 80% using a two-tailed Wilcoxon-Mann-Whitney test for two groups, a minimum of 367 fully completed responses in total (184 per group) should be included (see S3 File).

Schlesinger Group received the study information and a link to the questionnaire. They distributed both to their study panel. Detailed information on how the study was advertised or presented to the panel and how many people were invited is not available. Participants needed to be aged 18 years or older. Knowledge of the German language was required. The questionnaire translation in S4 File was based on a one way translation of German to English. The original questionnaire in German can be found in S5 File. Some text elements are still provided in German, as these are proper names of existing campaigns. Answering should not take longer than 15 minutes. Schlesinger group compensated the participants via a bonus point system. Quota management was only possible for gender and age. It was performed twice a day by CN using descriptive analysis of age and gender distribution. Recruiting started on March 29^th^, 2022. We decided to stop data collection after reaching 406 fully completed questionnaires. This target was reached on April 4^th^, 2022 after one week of data collection.

The software REDCap (Research Electronic Data Capture), hosted at Universitätsklinikum Erlangen, was used for programming the questionnaire as well as for data collection and management. REDCap is a secure, web-based software platform designed to support data capture for research studies [24,25]. The data were collected anonymously and saved on a secure server, hosted by Universitätsklinikum Erlangen. Data will be stored for ten years according to the requirements of the German Research Foundation. Participants needed to give their written informed consent before continuing. Participating was voluntary. The participants could end the survey at any time by closing the browser tab.

Ethical approval was granted by the Ethics Committee of the Faculty of Medicine of the Friedrich-Alexander University Erlangen-Nürnberg (21-434-S, 14.12.2021).

### Measures

The questionnaire was developed based on the results of a qualitative study [19] following the generalization model by Mayring [26]. The items of the questionnaire are based on participant’s answers of the qualitative study, experts’ opinions and findings of a literature research. [12,13,27].

At the beginning detailed information about the study’s objective, survey length and data handling were given in written form. The main part consisted of 45 questions about different topics: (1) utilization of health care services using single- and multiple-choice questions; (2) perception of overuse including understanding and estimation of prevalence using a four-point Likert scale (from “totally disagree” to “totally agree”) and relevance using single-choice question; (3) personal attitude to medical overuse including reasons, consequences and solutions using a four-point Likert-Scale (from “totally disagree” to “totally agree”). In the second part demographic and socio-economic characteristics were collected. To assess morbidity, the Self-Administered Comorbidity Questionnaire (SCQ-D) was used in a modified German version [28,29]. The original questionnaire as well as its translation to English are available as (S4 and S5 Files).

### Reasons for the chosen design

The participants were asked about their own definition of overuse at the beginning to avoid bias by giving them an official definition directly. The official definition given afterwards was meant to create a common ground for all participants. To prevent neutral responses, an even number of categories for the Likert-Scale questions was chosen [30]. Items were presented in random order when possible to avoid order effect bias [31]. Participants could not move back in the questionnaire to minimize social desirability [32]. All questions were programmed to be mandatory. Response pattern indices (semantic antonyms) were used to evaluate whether the questionnaire was filled out carefully [33,34]. The method was used within the items 16.1 and 16.2. No specific items like cookies or IP-addresses were included to enable identification of double data entry. A similarity check was performed using the inbuilt feature of IBM Statistics SPSS, Version 28.0.0.0. No identical data entries were found.

### Data analysis

Only fully answered questionnaires were included into the analysis. For statistical analysis the software IBM Statistics SPSS, version 28.0.0.0 was used.

Items on four-point Likert-Scales were considered as ordinally scaled [35]. Median and inter-quartile range (IQR) were computed. For graphical presentation, stacked bar plots were designed using statistic software R (Version 4.2.1) and R-Studio (Version 2022.07.0).

For the main topic “Perception of overuse” (26 questionnaire items) group differences based on gender and previous knowledge of overuse were assessed. The selection of gender was based on previous studies [36–39]. It was also assumed that the previous knowledge of overuse might influence peoples’ opinion. Mann-Whitney-U Test was used to compare groups. No adjustment for multiple testing was made due to the exploratory approach of the analysis. To report effect size, Pearson’s *r* was chosen [40]. Following Cohen’s recommendations, effect size of Pearson’s *r* is small if |*r*| > .*1*, medium if |*r*| > .3 and large if |*r*| > .5 [41]. All tests were considered significant with a p-value < .05. Answers to open-ended questions 8 and 9 were analyzed and then grouped into four categories: (1) “no definition given”, comprising answers like “I do not know” or random letters; (2) “wrong definition”, if they did not include anything related to medical overuse; (3) “more physicians than needed” comprising answers regarding a higher number of physicians than needed and (4) “Too much medicine, including overtesting and overtreatment”, included all answers, in which the participants mentioned too much medicine. We followed an inductive way of analysis and formed the categories during the analysis. Answers were coded by CN and revised by SH.

## Results

### Descriptive analysis

#### Study sample

A total of 540 people registered for the study. Of these 460 (85.2%) people agreed to participate and 406 (75.2%) fully completed the survey. No participants were excluded due to implausibility. Half of them were female (*n* = 210, 51.7%). About 40% were between 18 and 44 years old (*n* = 154, 37.9%), while about 30% each were between 45 and 64 years old (*n* = 127, 31.3%) or aged 65 or older (*n* = 125, 30.8%). The majority (*n* = 341, 84.0%) was insured in the statutory health insurance. Most respondents either had a professional training (*n* = 213, 52.5%) or a university degree (*n* = 177, 43.6%). Around two-thirds were employed (*n* = 273, 67.2%). Half of the participants lived in cities with more than 100.000 inhabitants (*n* = 198, 48.8%). On average, participants visited 2.46 physicians during the past three months (*SD* = 1.80). All sociodemographic characteristics are shown in Table 1. On average, participants stated they had 1.96 health problems (*SD* = 1.71). Back pain (*n* = 132, 32.5%) and hypertension (*n* = 130, 32.0%) were the most frequent (see S6 File for more detail).

**Table 1:** Socio-demographic characteristics and health care utilization.

	n	(%)
**Gender**		
Female	210	(51.7)
Male	195	(48.0)
Diverse	1	(0.2)
**Age groups**		
18 to 24 years	33	(8.1)
25 to 44 years	121	(29.8)
45 to 64 years	127	(31.3)
65 years or older	125	(30.8)
**Insurance status**		
Statutory health insurance	341	(84.0)
Private health insurance	65	(16.0)
**Professional/Vocational education**		
No professional training (yet)	16	(3.9)
Professional training	213	(52.5)
(technical) university degree	177	(43.6)
**Employment status**		
Not employed	10	(2.5)
In professional training / student	23	(5.7)
Employed	232	(57.1)
Self-employed	18	(4.4)
Retired	123	(30.3)
**Residence**		
Under 5,000 inhabitants	49	(12.1)
5,000 to 20,000 inhabitants	79	(19.5)
20,000 to 100,000 inhabitants	80	(19.7)
More than 100,000 inhabitants	198	(48.8)
**Who decides about patients’ treatment?** (***Range***: 0 = “Physician always decides”, 100 = “Patient always decides”; *M* = 49.56, *SD* = 17.97)		
Physician always decides (0–33 points)	51	(12.6)
Physician and patient decide together (34–66 points)	312	(76.8)
Patient always decides (67–100 points)	43	(10.6)
**Number of physicians visited per patient in the last three months** (*Median = 2*.*0*, *IQR = [1*.*0; 3*.*0]*)		
None	36	(8.9)
One or two	191	(47.0)
Three or more	179	(44.1)

#### Understanding of medical overuse

A majority (*M* = 237, 58.4%) had never heard of medical overuse before. After being presented the official definition, 263 (64.8%) stated they had not previously experienced it. When asked in which two areas of treatments they suspected medical overuse most, approximately half of all stated self-pay services (*n* = 229, 56.4%), surgical interventions (*n* = 184, 45.3%) and prescriptions of drugs (*n* = 151, 37.2%). Only a small number of participants suspected overuse most in early detection and screening (*n* = 32, 7.9%) or blood tests (*n* = 43, 10.6%). In the open questions, 246 (60.6%) described overuse as too much medicine, including overtesting and overtreatment. About half of the participants thought that less overuse would improve the health care system (*n* = 201, 49.5%). All results can be found in Table 2.

**Table 2:** Awareness of medical overuse.

	n	(%)
**Have you ever heard of medical overuse before?**		
Yes	169	(41.6)
No	237	(58.4)
**Have you already experienced or perceived overuse according to this definition?**		
Yes	143	(35.2)
No	263	(64.8)
**Understanding of medical overuse**		
“Too much medicine, incl. overtesting and overtreatment”	246	(60.6)
“More physicians than needed”	59	(14.5)
Wrong definition	16	(3.9)
No definition given	85	(20.9)
**Areas connected to medical overuse (two items could be chosen)**		
Self-pay services	229	(56.4)
Surgeries	184	(45.3)
Prescription of medicine	151	(37.2)
Imaging procedures	90	(22.2)
Blood tests	43	(10.6)
Early detection and screening	32	(7.9)
**Relevance of medical overuse in the German healthcare system**		
“Less medical overuse would improve our health system.”	201	(49.5)
“There are other issues that need to be addressed.”	98	(24.1)
“Medical overuse exists but it does not have a negative impact on health care provision.”	62	(15.3)
“Medical overuse does not exist in our health system.”	45	(11.1)

**Fig 1:**
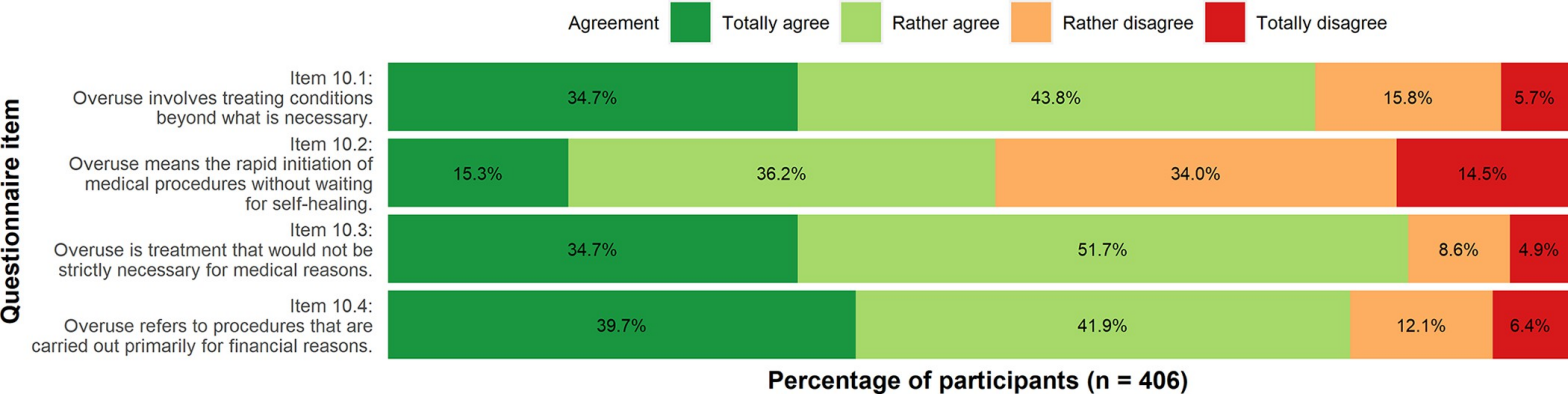
Aspects connected to medical overuse. Descriptive analysis.

There was broad agreement that overuse involved treatment beyond necessity (Item 10.1, 34.7% totally agree, 43.8% rather agree; Item 10.3, 34.7% totally agree, 51.7% rather agree). The participants also agreed that overuse refers to procedures which are carried out due to financial reasons (Item 10.4, 39.7% totally agree, 41.9% rather agree). All results are depicted in Fig 1.

#### Perception of medical overuse

**Reasons**: There was a high degree of agreement that the following items contribute to overuse: availability of medical equipment (Item 15.6, 29.8% totally agree, 52.5% rather agree), reimbursement of medical services (Item 15.8, 30.8% totally agree, 50.7% rather agree) and missing coordination between providers (Item 15.7, 30.5% totally agree, 52.2% rather agree). In diagnostics and therapy, the majority of participants seem to expect action rather than a wait-and-see attitude (Item 15.2, 55.2% rather agree; Item 15.3, 60.1% rather agree). The participants tended to agree that patients themselves are requesting medical services (Item 15.10, 24.1% totally agree, 58.1% rather agree).

**Consequences**: Participants mostly agreed that medical overuse may lead to rising health care costs (Item 16.6, 40.1% totally agree, 45.6% rather agree). Less total agreement occurred in the responses to the question of whether medical care could endanger patients’ physical or and mental well-being (Item 16.3, 14.5% totally agree; item 16.4, 16.0% totally agree).

**Fig 2:**
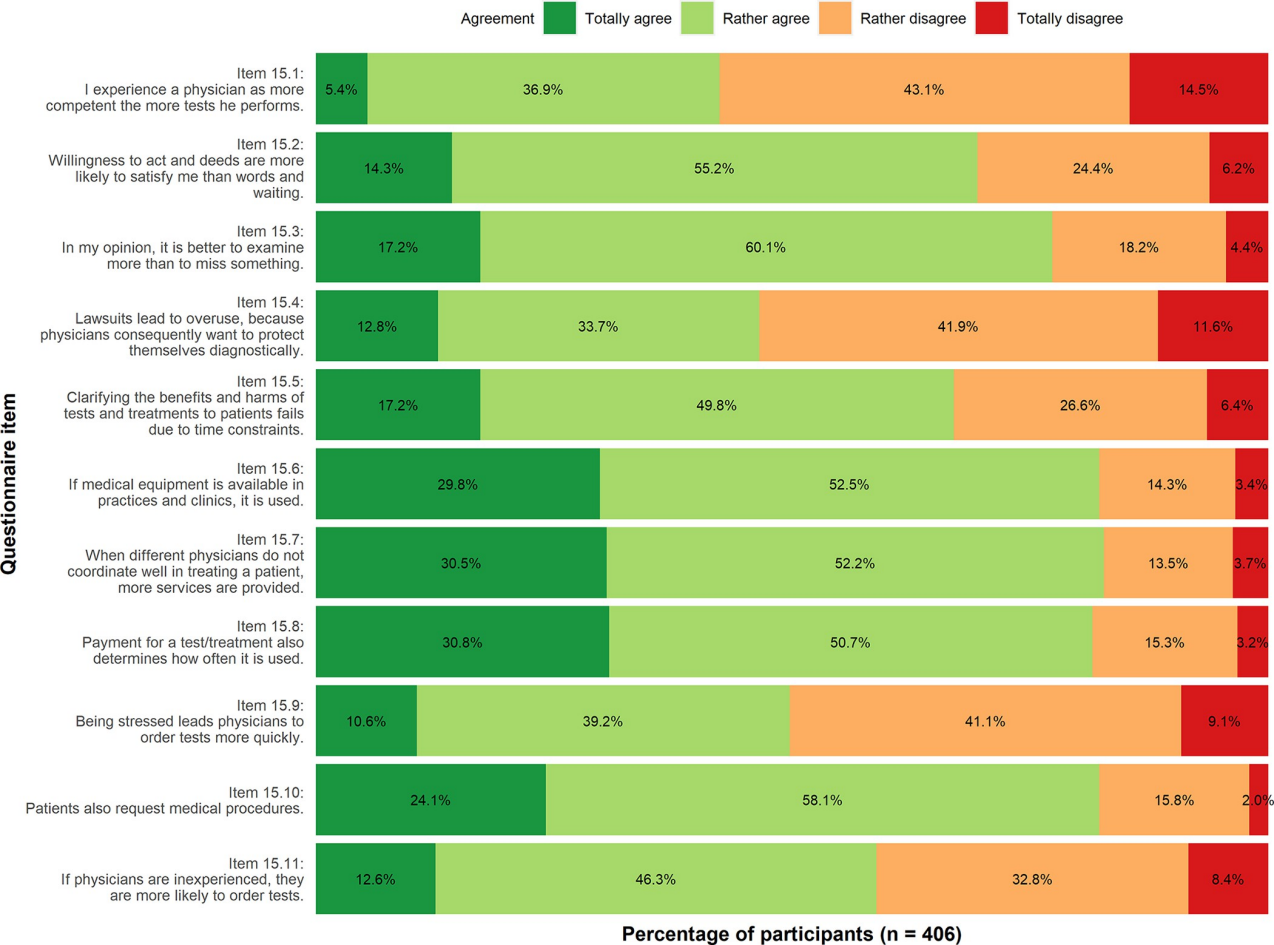
Reasons of medical overuse. Descriptive analysis.

**Fig 3:**
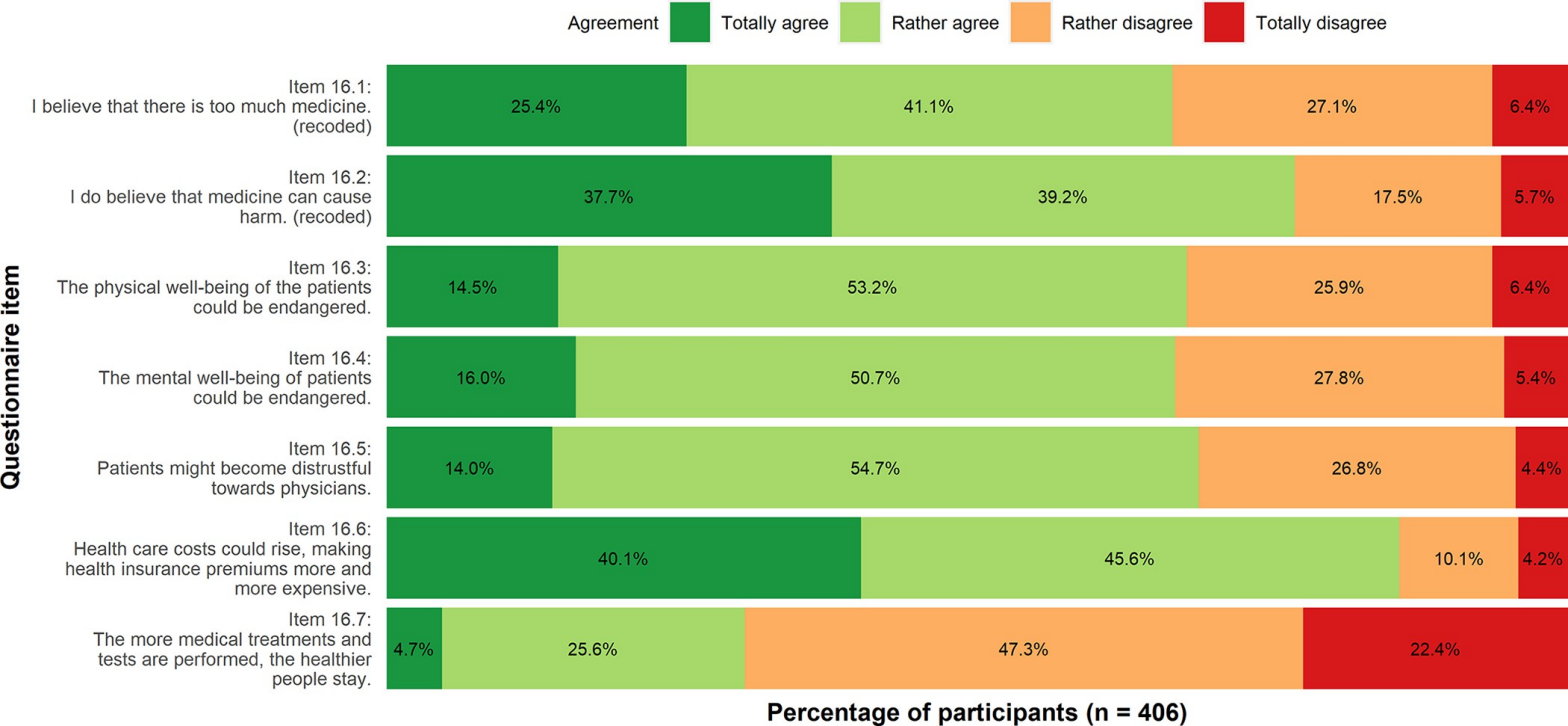
Consequences of medical overuse. Descriptive analysis.

**Fig 4:**
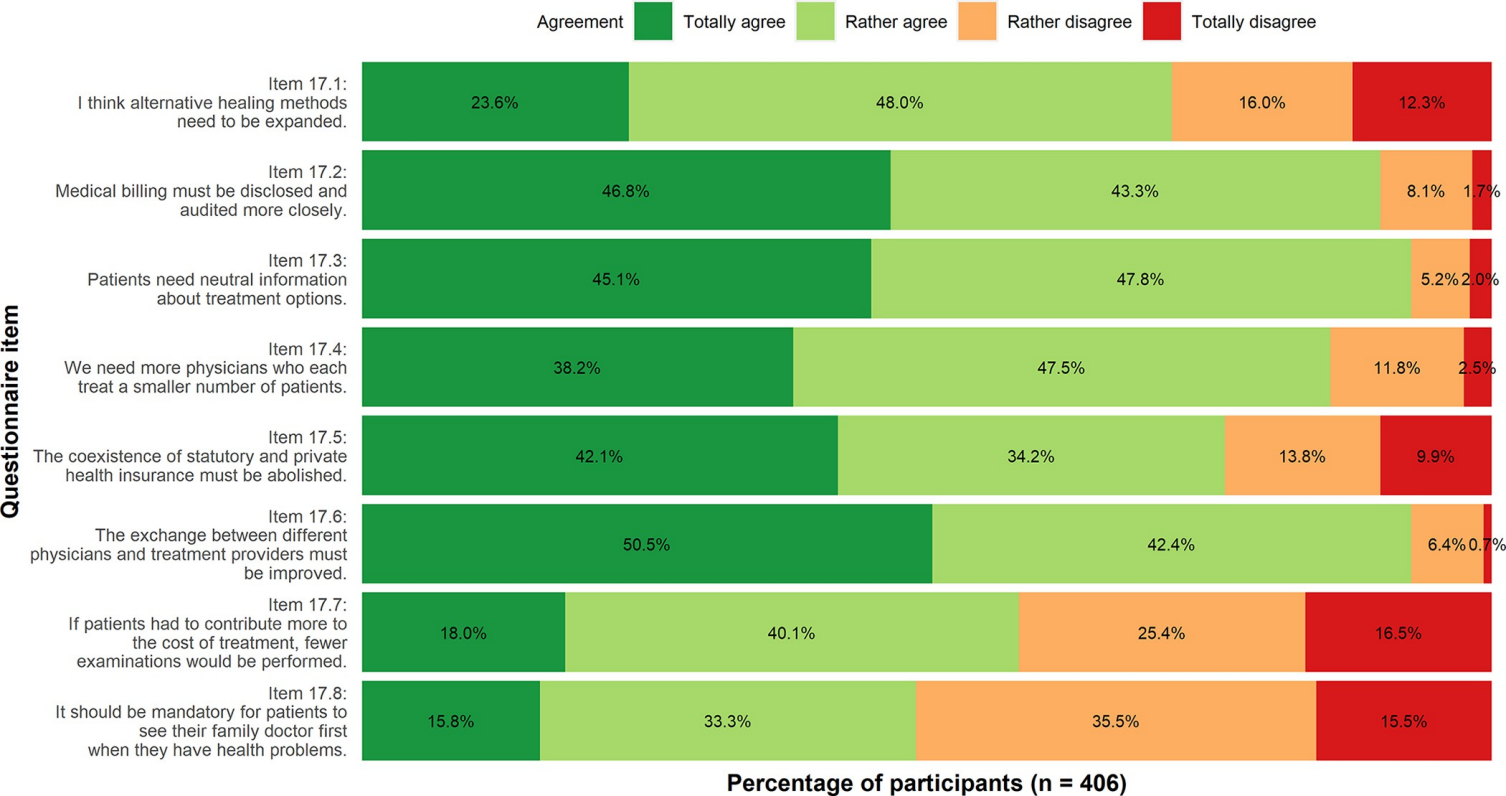
Approaches to reduce medical overuse. Descriptive analysis.

**Solutions**: Disclosing medical billing (Item 17.2, 46.8% totally agree, 43.3% rather agree), abolishing the coexistence of statutory and private health insurance (Item 17.5, 42.1% totally agree, 34.2% rather agree*)* and providing patients with neutral health information (Item 17.3, 45.1% totally agree, 47.8% rather agree) were seen as possible solutions. Also, more physicians treating less patients each (Item 17.4, 38.2% totally agree, 47.5% rather agree) and a better cooperation between health care providers were seen as required (Item 17.6, 50.5% totally agree, 42.4% rather agree). However, fewer participants agreed that patients should contribute more to reduce health care costs (Item 17.7, 18.0% totally agree, 40.1% rather agree). The statement that patients should see their GP first also had less agreement (Item 17.8, 15.8% totally agree, 33.3% rather agree). Figs 2–4 show the results in detail.

Existing campaigns to reduce overuse were hardly known (n = 326, 80.3%). The most known campaign was “Less is more” (n = 41, 10.1%; Klug entscheiden: n = 29, 7.1%; Smarter Medicine: n = 19, 4.7%; Quartäre Prävention; n = 15, 3.7%; Choosing Wisely: n = 11, 2.7%). Multiple campaigns could be chosen.

### Group differences

**Gender:** Female participants showed higher agreement than males with reasons and solutions for reducing medical overuse that related to structural aspects of the health care system. These are time constraints leading to less information for patients as a reason (Item 15.5: *p* = .008*, *r* = -.13) and increasing the number of physicians (Item 17.4: *p* = .008*, *r* = -.13) or abolishing the coexistence of the statutory and private health insurance (Item 17.5: *p* = .003*, *r* = -.15) as solutions. However, male participants see reasons for and solutions to reduce medical overuse more in items related to financial aspects like lawsuits as reason (Item 15.4: *p* = .010*, *r* = -.13) and higher contribution to treatment costs (Item 17.7: *p* < .001*, *r* = -.19) as solution than females. **Previous knowledge of overuse:** Participants who knew about medical overuse prior to the study showed a higher acceptance of financial reasons for medical overuse such as lawsuits (Item 15.4: *p* = .006*, *r* = -.14), availability of medical equipment (Item 15.6: *p* < .001*, *r* = -.20), payment for medical treatment (Item 15.8: *p* = .003*, *r* = -.15) than participants without said knowledge. They also showed higher agreement to solutions to reduce medical overuse related to financial aspects such as disclosure of medical bills (Item 17.2: *p* = .002*, *r* = .16) or higher patient contributions to treatment costs (Item 17.7: *p* = .008*, *r* = .13).

Results of group differences are in S7 File.

## Discussion

### Key findings

Our aim was to assess the understanding of medical overuse in the German population, what people presume as reasons and consequences and what solutions they would prefer. An online survey was conducted, distributed via an institute for market research in March/April 2022. The term medical overuse was not familiar to many respondents. If asked, most participants stated that medical overuse, including overtesting, could be seen as treatment beyond medical necessity and medical procedures carried out mainly due to financial reasons. Nearly half agreed that reducing overuse would help to improve the healthcare system. Overuse was mainly suspected in self-pay services, surgeries and prescriptions of drugs but considerably less in diagnostic tests and screening. Participants suspected privately insured citizens to be more affected by overuse than citizens with statutory health insurance. Reasons were attributed to the availability of medical equipment, reimbursement for medical services for physicians and missing coordination between health care providers. The majority thought of medical overuse being a reason of rising health care costs and rising insurance premiums. Harms to patients were rated less of a problem. Structural changes as abolishing the coexistence of statutory and private health insurance, limiting the numbers of patients seen by physicians and disclosing medical billing were seen as an effective way to reduce medical overuse. Existing campaigns aiming to reduce medical overuse were barely known. Previous knowledge of overuse did affect the participants’ tendency to identify components and reasons of overuse.

### Discussion of findings

Similar to other research findings, our participants tended to have a vague and superficial understanding of the concept “medical overuse” [16,19,42,43]. They seemed to immediately understand the high relevance, but as in other studies did not perceive themselves being affected by medical overuse [16,19]. Participants were more satisfied with medical action than with waiting for symptoms to resolve naturally. As a potential driver, the desire to act instead of a watchful waiting approach as well as the belief that more care is better than less was also mentioned by Morgan et al. (2015). Our findings correspond well to the literature in that financial incentives are seen as major drivers [4,16–18,44]. Other studies also mentioned defensive medicine and to avoid harmful consequences for patients as reasons for overuse [4,16–18,44,45]. In contrast, physical and psychological harms as well as increasing distrust towards physicians were seen as a minor problem. A systematic literature review and a meta-synthesis correspond to these findings [16,43]. Especially cancer screening almost inevitably is leading to overdiagnosis, but our participants seemed not to identify screening services and early detection as being associated with medical overuse as often as other medical procedures. How should they, when these services are even payed for and promoted by health insurance companies and other stakeholders in Germany? Risks of screening are often neglected, most likely because people predominantly believe that early detection saves lives [14,43].

A large part of the German population supports the idea of abolishing the coexistence of statutory and private health insurance [46]. However, the current political agenda of the elected parties (coalition agreement) no longer includes the topic [47]. Another approach seems to be the alignment of the reimbursement schemes between statutory and private health insurance for outpatient care. The aim would be to diminish financial incentives in overtreatment of privately insured patients [37–39,48]. Physicians argue that the higher reimbursement for privately insured patients compensates the inappropriately low reimbursements for publicly insured patients. Therefore aligning the two schemes is discussed critically [49,50]. Another suggested approach might be to disclose medical billing to the patients and to audit billing more strictly. In the German healthcare system, medical services are delivered to patients widely without copayments or deductibles. Medical bills for publicly insured patients are paid directly by the insurer. These patients, which are by far the majority, rarely know the costs of their treatment [37]. Increasing the patients’ contribution to the costs of care could be an approach to reduce patient-driven overuse [51] but it could also lead to patients avoiding medical services due to financial burdens [52].

Studies indicate that time constraints and inexperience in shared decision-making might also be drivers of medical overuse [4,17–19,44]. Our results showed that people seemed to be aware of it but that they did not rate it as a major cause. The participants mentioned limiting the number of patients for each physician as a promising approach to reduce medical overuse. Germany, however, already has a high number of physicians per 1.000 inhabitants in comparison to other OECD countries. Since the year 2000, the number has even risen by about 40% [53]. But the number of full-time equivalents has been declining for years, meaning that more physicians overall spend less time treating patients [54,55]. Increasing the number of physicians could be an approach but faces the problem of impending shortage of physicians. Also, as long as reimbursement is tied to medical action, physicians might even increase the number of medical procedures for these fewer patient to maintain their revenues.

Improving citizens’ knowledge might be a solution. Citizens wish to have more neutral health information, as our and other studies show [16,19]. Campaigns like “Choosing Wisely” or “Klug entscheiden”, which are meant to provide neutral information, have been established but are not widely known in the population [19,44]. More commonly, citizens increasingly tend to use the internet to inform themselves [56,57]. Quality and reliability of online information, however, can vary from professionally reviewed to personally reported information in blogs to disease mongering driven by financial interests. Citizens might lack the ability to evaluate these correctly and thus become misinformed, more afraid and uncertain about appropriateness of care. Misinformed citizens might request unnecessary care [16,56,57]. Raising public awareness of reliable sources might be a first step towards providing citizens with understandable and correct information.

Improving the coordination between health care providers would presumably help reduce medical overuse in the participants’ opinion. In Germany, especially inter-sectional interfaces of health care lack coordination. Our participants rated examinations being carried out multiple times by different physicians as drivers for overuse [19,58,59]. Other reasons are seen in time constraints, high numbers of patients per physician and legal regulations as well as the low amount of digitalization in the German healthcare system [58]. Improving digitalization might bear the potential to reduce health care expenditure and unnecessary care [60]. Electronic systems sharing health care information between all health care sectors are inexistent [61].

Another cause for difficulties in the coordination between different physicians could also be the so-called dual physician track in Germany, a euphemism for the fact that many physicians are caring for the same patients in parallel [37]. Citizens are almost free to choose the physician they wish to see. Our study participants did not seem to think that implementing a primary care based system would be an appropriate approach to reduce overuse. In the statutory health insurance scheme in Germany, it is possible to choose a gate-keeping program on voluntary basis, called “general practitioner (GP) -centered care” [37] Only a small amount of the insured actually takes part in the GP-centered care, but a positive trend has been observed [62]. This leads to the conclusion that people see problems in care coordination but would rather not restrict their own freedom in the choice of their physician.

A reason for females agreeing more to items related to structural reasons for and solutions to reduce medical overuse might be that they tend to visit doctors more often (because of their own complaints or as companions for children or those in need of care) [63,64] and are thus more likely to perceive structural problems in the health care system than men. Our findings show that knowledge about the concept of medical overuse is linked to a stronger connection with financial causes, consequences and possible solutions. Other studies also show similar trends [19]. The reason could be the frequently discussed scarce financial resources in the German health care system [65] as well as the significant differences in remuneration between the health insurance systems, which may lead to a preference for financially lucrative services and patients [37–39,48].

### Strengths and limitations

Our study was the first in Germany to evaluate citizens understanding of medical overuse in a quantitative design. The questionnaire was designed systematically following the results of different qualitative analyses [12,13,19,27]. The questionnaire was tested in a pre-test. The pre-test was conducted to evaluate the design and technical functionality as well as the comprehensibility and relevance of the questionnaire. Validity and reliability measurements were not included. The study design only allowed assessing the participants’ attitudes towards medical overuse but not their actual behavior. However, within limits it is possible to draw conclusions from attitude to behavior. We did not perform any specific methods in sample collection that would guarantee representativeness of our results. However, age and gender distribution as well as the distribution of the participants’ employment status almost met the real distribution in Germany. Privately insured persons were slightly overrepresented. The percentage of participants with university degree was slightly more than twice as high as the percentage in the German population. People with no professional education (yet) were underrepresented. It was not possible to steer data collection according to these criteria. The results only showed effect sizes in a range from .13 to .20. We estimated an effect size ranging from .20 to .30 and calculated the needed sample size accordingly. Further studies should probably make the assumption that sociodemographic characteristics might only account for marginal differences in peoples’ attitudes and therefore would require a larger sample size.

The survey was designed as an online survey. Participation therefore required certain technical skills and the availability of technical devices and internet access. An institute of market research carried out the data collection. Their study panels might limit the representativeness of the results. The participants also received an incentive in form of bonus points for completing the survey. It is possible that they answered the questionnaire not carefully to complete it in a short amount of time and receive the incentive. Answers were checked for implausible patterns. No identifiers were included to enable checking for multiple data entry. IBM Statistics SPSS allowed performing a similarity check, which did not indicate any identical data. Inspecting the free text questions showed highly similar responses which might indicate that some individuals filled out the questionnaire more than once. We therefore cannot exclude double data entry. In the open-ended question about the participants’ definition of overuse, it must be remembered that these could only be answered in short key points. There was no in-depth analysis to assess the participants’ understanding of the term “medical overuse”. It was furthermore not possible to check whether an internet search was carried out to answer the questions.

The survey was only presented in German language. We advised participants not to have the website translated automatically, as this can lead to errors in the translation. However, we could not check whether this advice was listened to. We also could not check whether the participants had sufficient knowledge of the German language to understand the questionnaire correctly. Misunderstandings can therefore not be ruled out.

Nonetheless, findings should be interpreted with caution as generalization might be reduced. The voluntary participation can lead to a potential bias as it can be assumed that mostly citizens who are interested in medical overuse and research in general might have been more likely to respond to our questionnaire, skewing the sample.

## Conclusion

Citizens in Germany are little familiar with the term “medical overuse” but seem to grasp its meaning immediately. They tend to link medical overuse mainly to financial incentives. Medical overuse was suspected more in medical procedures with high remuneration and less in imaging or screening procedures. Respondents seemed to rate medical action positively and underestimate the physical and psychological harms possibly resulting from it. Patients were identified as potential causes for medical overuse, but the participants did not include themselves in the group of patients who drive overuse. Approaches to avoid medical overuse are only seen in reducing financial incentives and redesigning the German health insurance schemes. The idea of implementing a primary health care system gets little agreement. Increasing the awareness of medical overuse might be an important step forward. Awareness of the direct harms of medical overuse for patients should be strengthened.

## Supporting information

S1 Fig Sample size calculation. (TIF)

S1 Table Study data. (CSV)

S1 File STROBE statement. (DOCX)

S2 File CHERRIES checklist. (DOCX)

S3 File Study questionnaire (English version). (DOCX)

S4 File Study questionnaire (German version). (DOCX)

S5 File Health problems mentioned by study participants. (DOCX)

S6 File Results of group analysis. (DOCX)

S7 File Code of analysis. (SPS)

S8 File Codebook. (PDF)
