# Supplementary figures and images for: What do people know and think about medical overuse? an online questionnaire study in Germany
Source: PLoS One. 2024 Mar 7;19(3):e0299907. doi: 10.1371/journal.pone.0299907 (PMC10919641; doi:10.1371/journal.pone.0299907)

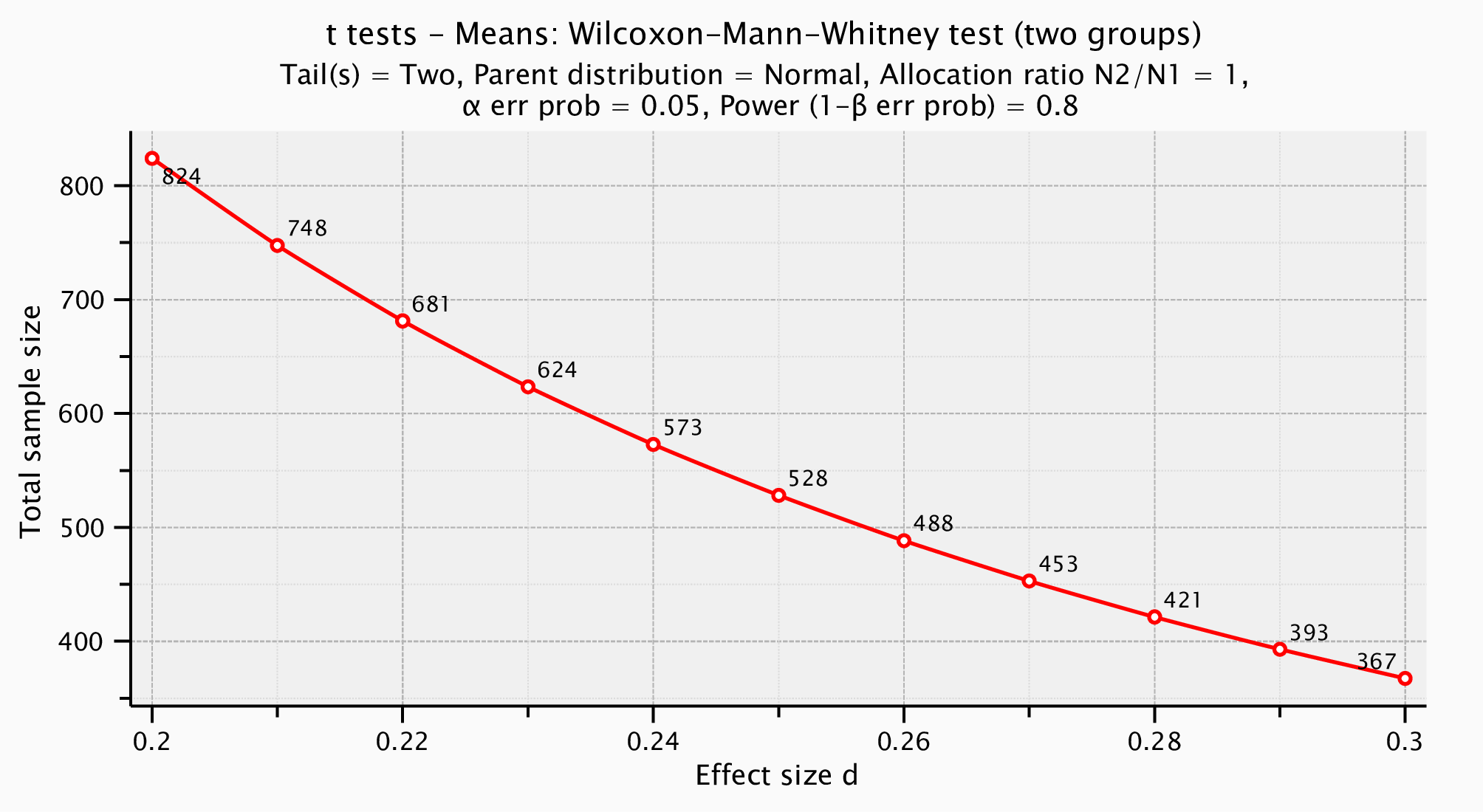

Supplement: S1 Fig — (TIF) [file pone.0299907.s001.tif]
