# Supplementary material for: What do people know and think about medical overuse? an online questionnaire study in Germany
Source: PLoS One. 2024 Mar 7;19(3):e0299907. doi: 10.1371/journal.pone.0299907 (PMC10919641; doi:10.1371/journal.pone.0299907)
Supplement: S3 File — (DOCX) [file pone.0299907.s005.docx]

# S4 File. Study questionnaire (English version).

## Study on overuse

### Registration

Dear Sir or Madam,

Thank you for finding the time to participate in our survey on "Perceptions of Overuse".

**The project**

What is it about? We are interested in your opinion on the topic of "overuse in medicine", how you understand it, when and where you notice it, what reasons you suspect for overuse and which potential solutions you see for it.

Who is conducting this study? The Institute of General Practice at the Universitätsklinikum Erlangen.

**Data security**

Important information on data protection:

Your data will be processed anonymously. No information is requested via which your answers can be assigned to your person. The data will be stored in electronic form on a secure server at the Universitätsklinikum Erlangen. Access is only granted to scientists of the Institute of General Practice who are involved in the project. The data are secured against unauthorized access. After the completion of the study, the collected data will be stored for 10 years according to the regulations of the German Research Foundation. All information obtained is subject to the provisions of the Federal Data Protection Act (BDSG) and the Bavarian Data Protection Act (BayDSG).

**Right of withdrawal**

Participation in the survey is voluntary. You can end the survey at any time by closing the browser window. Your data will then not be included in the evaluation. Data that has already been submitted cannot be subsequently revoked due to the anonymous collection.

Filling out the questionnaire takes about 10-15 minutes. Please answer the individual questions spontaneously and according to your personal assessment. **There is no "right" or "wrong".**

**Please do not use automatic language translation. Experience has shown that errors occur in the translation.**

For better readability, we use the shorter, masculine form. Of course, we address all genders equally.

**Please note that you must answer all questions in order to continue in the questionnaire. If you have missed a question, a note will appear to alert you to the missing answer.**

1. I declare my consent to participate in the study and to the storage of my answers. I agree with the privacy policy.
   - Yes *(PROG: 4.)*
   - No *(PROG: 2.)*
2. Thank you for showing interest in our survey. Without your consent to the participation and privacy policy, participation is unfortunately not possible. You can change your selection again or end the survey here.
   - I would like to change my consent (PROG: 3.)
   - I would now like to end the questioning. (PROG: END)
3. I give my consent to participate in the study and to the storage of my answers. I agree with the privacy policy
   - Yes *(PROG: 4.)*

### Utilization of health care services

#### Please think about your own visits to the physician and your own health behavior

1. Are you insured under the statutory or private health insurance scheme?
   - Statutory health insurance
   - Private health insurance
2. Which physicians have you seen in the last three months?
   - No visit to the physician
   - Family doctor
   - Internist (e.g. cardiologist, gastroenterologist, ...)
   - Oncologist
   - Orthopedist
   - Urologist
   - Gynecologist
   - Radiologist
   - Psychiatrist or psychotherapist
   - Ear, nose and throat specialist
   - Dermatologist
   - Neurologist
   - Dentist or oral surgeon
   - Eye specialist
   - Other specialty
3. How are decisions about treatment and tests made during your appointments with your physician?

| Decision is made  by the physician | Decision is made by the physician and myself jointly | Decision is made  only by myself |
| --- | --- | --- |

### Perception of overuse

#### Please provide some information on whether and what you have already heard about overuse and how you understand it.

1. Have you ever heard of medical overuse?
   - Yes *(PROG: 8.)*
   - No *(PROG: 9.)*
2. What do you think overuse means?

_______________________________

*Please answer in short key points*

1. What do you think could be meant with overuse?

_______________________________

*Please answer in short key points*

#### Overuse is described in a number of ways

1. How much do you agree with the following statements?

|  |  | Totally  disagree | Rather  disagree | Rather  agree | Totally  agree |
| --- | --- | --- | --- | --- | --- |
| 10.1. | Overuse involves treating conditions beyond what is necessary. |  |  |  |  |
| 10.2. | Overuse means the rapid initiation of medical procedures without waiting for self-healing. |  |  |  |  |
| 10.3. | Overuse is treatment that would not be strictly necessary for medical reasons. |  |  |  |  |
| 10.4. | Overuse refers to procedures that are carried out primarily for financial reasons. |  |  |  |  |

#### In the German health care system, overuse is defined as "care that exceeds the individual needs of the patient. This includes services without benefit for the patient".

1. Have you already experienced or perceived overuse according to this definition?
   - Yes
   - No
2. How would you rate the importance of overuse? *Please select the answer you agree with the most.*
   - Less medical overuse would improve our health system.
   - There are other issues that need to be addressed.
   - Medical overuse exists but it does not have a negative impact on healthcare provision.
   - Medical overuse does not exist in our health system.
3. In which medical services do you suspect overuse? *Please mark the two areas in which you most frequently suspect overuse.*
   - Individual health services (IGeL)
   - Surgeries
   - Prescription of medicine
   - Imaging procedures (e.g. ultrasound, X-ray, …)
   - Blood tests
   - Early detection and screening (e.g. cancer screening)
4. Please indicate which group of people you suspect are more likely to be affected by overuse?

|  |  | Totally  disagree | Rather  disagree | Rather  agree | Totally  agree |
| --- | --- | --- | --- | --- | --- |
| 14.1. | People with private health insurance are more often affected than those with statutory health insurance. |  |  |  |  |
| 14.2. | People with a higher level of education are more often affected than people with a lower level of education. |  |  |  |  |
| 14.3. | People with higher income are more often affected than people with lower income. |  |  |  |  |
| 14.4. | Younger people are more often affected than older people (e.g. pensioners). |  |  |  |  |

### Reasons for overuse

#### Decisions for or against a diagnostic measure or medical treatment are determined by many factors.

1. Where do you rather suspect causes for too much medicine?

|  |  | Totally  disagree | Rather  disagree | Rather  agree | Totally  agree |
| --- | --- | --- | --- | --- | --- |
| 15.1. | I perceive a physician as more competent the more tests he performs. |  |  |  |  |
| 15.2. | Willingness to act and actions are more likely to satisfy me than words and waiting. |  |  |  |  |
| 15.3. | In my opinion, it is better to examine more than to miss something. |  |  |  |  |
| 15.4. | Lawsuits lead to overuse, because physicians consequently want to protect themselves diagnostically. |  |  |  |  |
| 15.5. | Clarifying the benefits and harms of tests and treatments to patients fails due to time constraints. |  |  |  |  |
| 15.6. | If medical equipment is available in practices and clinics, it is used. |  |  |  |  |
| 15.7. | When different physicians do not coordinate well in treating a patient, more services are provided. |  |  |  |  |
| 15.8. | Payment for a diagnostic measure/treatment also determines how often it is used. |  |  |  |  |
| 15.9. | Being stressed leads physicians to order tests more quickly. |  |  |  |  |
| 15.10. | Patients also request medical procedures. |  |  |  |  |
| 15.11. | If physicians are inexperienced, they are more likely to order tests. |  |  |  |  |

### Consequences of overuse

#### Excessive treatment beyond the patient’s benefit has various consequences.

1. What do you suspect might be consequences of too much medicine and overuse?

|  |  | Totally  disagree | Rather  disagree | Rather  agree | Totally  agree |
| --- | --- | --- | --- | --- | --- |
| 16.1. | I cannot imagine at all that there is really too much medicine. |  |  |  |  |
| 16.2. | I do not believe that medicine can cause harm. |  |  |  |  |
| 16.3 | The physical well-being of the patients could be endangered. |  |  |  |  |
| 16.4. | The mental well-being of patients could be endangered. |  |  |  |  |
| 16.5. | Patients might become distrustful towards physicians. |  |  |  |  |
| 16.6. | Health care costs could rise, making health insurance premiums more and more expensive. |  |  |  |  |
| 16.7. | The more medical treatments and tests are performed, the healthier people stay. |  |  |  |  |

### Approaches to reduce or avoid overuse

1. What measures do you think could contribute to appropriate care?

|  |  | Totally  disagree | Rather  disagree | Rather  agree | Totally  agree |
| --- | --- | --- | --- | --- | --- |
| 17.1. | I think alternative healing methods need to be expanded. |  |  |  |  |
| 17.2. | Medical billing must be disclosed and audited more closely. |  |  |  |  |
| 17.3 | Patients need neutral information about treatment options. |  |  |  |  |
| 17.4. | We need more physicians who each treat a smaller number of patients. |  |  |  |  |
| 17.5. | The coexistence of statutory and private health insurance must be abolished. |  |  |  |  |
| 17.6. | The exchange between different physicians and treatment providers must be improved. |  |  |  |  |
| 17.7. | If patients had to contribute more to the cost of treatment, fewer examinations would be performed. |  |  |  |  |
| 17.8. | It should be mandatory for patients to see their family physician first when they have health problems. |  |  |  |  |

1. The public is beginning to address overuse in a variety of ways. Which of the following campaigns have you heard of?
   - “Choosing Wisely”
   - “Less is more”
   - “Klug entscheiden”
   - “Smarter Medicine”
   - “Quartäre Prävention”
   - None of the above
2. Please think about the health care system in 10 years. Do you think it will be better or worse than it is now?
   - System will be significantly worse
   - System will be slightly worse
   - System will be just as good as now
   - System will be slightly better
   - System will be significantly better

### Demographics

#### At the end of the survey, we would ask you to provide some more information about yourself and your background.

1. Please enter your gender
   - Female
   - Male
   - Divers
2. Please indicate which age group you belong to.
   - 18 to 24 years
   - 25 to 44 years
   - 45 to 64 years
   - 65 years or older
3. What is your highest professional qualification?
   - No professional training (yet)
   - Professional training
   - (Technical) university degree (Bachelor, Master, Diploma, ...)
4. Please indicate the status of your employment. *Employment is understood to mean any paid activity.*
   - Not employed
   - In professional training/student
   - Employed (as employee/worker, civil servant, including mini-job)
   - Self-employed
   - Retired
5. How many inhabitants does your place of residence have?
   - Under 5,000
   - 5,000-20,000
   - 20,000-100,000
   - Over 100,000

### Morbidity and health behavior

#### At the end of the survey, we would ask you to provide some more information about yourself and your background.

1. Which of the following health problems do you suffer from?
   - Heart disease
   - Hypertension
   - Lung disease
   - Diabetes
   - Gastrointestinal disease
   - Kidney disease
   - Liver disease
   - Anemia
   - Coagulation problems (e.g. thrombosis, embolism)
   - Cancer
   - Depression
   - Arthrosis
   - Back pain
   - Rheumatism or autoimmune disease
   - Allergies
   - Thyroid disease (hyper- or hypothyroidism)
   - None of the above complaints (PROG: 27)
   - No health problems (PROG: 27)
2. Please tick which of the previously selected complaints are chronic, for which complaints you regularly take medication and for which complaints you regularly go to the physician. *You can tick more than one answer per complaint. Please tick "None of the above" if none of the three statements apply.*

|  |  | It is a chronic disease. | I take medication regularly because of it. | I go to the physician regularly because of it. | None of the above |
| --- | --- | --- | --- | --- | --- |
| 26.1. | Heart problems |  |  |  |  |
| 26.2. | Hypertension |  |  |  |  |
| 26.3 | Lung problems |  |  |  |  |
| 26.4. | Diabetes |  |  |  |  |
| 26.5. | Gastrointestinal problems |  |  |  |  |
| 26.6. | Kidney problems |  |  |  |  |
| 26.7. | Liver problems |  |  |  |  |
| 26.8. | Anemia |  |  |  |  |
| 26.9. | Coagulation disorder (e.g. thrombosis, embolism) |  |  |  |  |
| 26.10. | Cancer |  |  |  |  |
| 26.11. | Depression |  |  |  |  |
| 26.12. | Arthrosis |  |  |  |  |
| 26.13. | Back pain |  |  |  |  |
| 26.14. | Rheumatism or autoimmune disorder |  |  |  |  |
| 26.15. | Allergies |  |  |  |  |
| 26.16 | Thyroid disease (hyper- or hypothyroidism) |  |  |  |  |

### End of the survey

Thank you for participating in our survey. With your help, we will gain a better understanding of what the population understands by medical overuse and where to start in order to reduce and avoid the phenomenon.

1. If you would like to make any further comments, you now have the opportunity to do so. Please use the following window for this.

_______________________________

*Please answer in short key points*

The results of the study are expected to be published on the website of the General Medicine Institute of the Universitätsklinikum Erlangen in August or September 2022.
