# Supplementary material for: What do people know and think about medical overuse? an online questionnaire study in Germany
Source: PLoS One. 2024 Mar 7;19(3):e0299907. doi: 10.1371/journal.pone.0299907 (PMC10919641; doi:10.1371/journal.pone.0299907)
Supplement: S4 File — (DOCX) [file pone.0299907.s006.docx]

# S5 File. Study questionnaire (German version).

## Studie zu Überversorgung

### Registrierung

Sehr geehrte Damen und Herren,

vielen Dank, dass Sie die Zeit finden, an unserer Studie „Wahrnehmung von Überversorgung“ teilzunehmen.

**Das Projekt**

Worum geht es? Uns interessiert Ihre Meinung zum Thema „Überversorgung in der Medizin“, was Sie darunter verstehen, wo Sie es wahrnehmen, welche Ursachen Sie vermuten und wo Sie potentielle Lösungen dafür sehen.

Wer führt diese Studie durch? Das Allgemeinmedizinische Institut des Universitätsklinikums Erlangen.

**Datenschutz**

Wichtige Informationen zum Datenschutz:

Ihre Daten werden anonymisiert verarbeitet. Es werden keine Informationen abgefragt, über die Ihre Antworten Ihrer Person zugeordnet werden können. Die Daten werden in elektronischer Form auf einem Server des Universitätsklinikums Erlangen gespeichert. Zugriff erhalten nur am Projekt beteiligte Wissenschaftler des Allgemeinmedizinischen Instituts. Die Daten sind gegen unbefugten Zugriff gesichert. Nach Beendigung der Studie werden die erhobenen Daten entsprechend den Vorgaben der Deutschen Forschungsgemeinschaft 10 Jahre gespeichert. Alle gewonnenen Informationen unterliegen den Bestimmungen des Bundesdatenschutzgesetzes (BDSG) und des Bayerischen Datenschutzgesetzes (BayDSG).

**Rücktrittsrecht**

Die Teilnahme an der Befragung ist freiwillig. Sie können die Befragung jederzeit durch Schließen des Browserfensters beenden. Ihre Angaben werden dann nicht in die Auswertung eingeschlossen. Bereits erfasste Daten sind nachträglich durch die anonymisierte Erhebung nicht mehr widerrufbar.

Das Ausfüllen des Fragebogens dauert ca. 10-15 Minuten. Bitte beantworten Sie die einzelnen Fragen spontan und nach Ihrer persönlichen Einschätzung. **Es gibt kein „Richtig“ oder „Falsch“.**

**Bitte verwenden Sie keine automatische Sprachübersetzung. Erfahrungsgemäß treten hier Fehler in der Übersetzung auf.**

Zur besseren Lesbarkeit verwenden wir die kürzere, männliche Form. Selbstverständlich sprechen wir alle Geschlechter gleichberechtigt an.

**Bitte beachten Sie, dass Sie alle Fragen beantworten müssen, um im Fragebogen fortfahren zu können. Haben Sie eine Frage übersehen, so erscheint ein Hinweis, der Sie auf die fehlende Antwort aufmerksam macht.**

1. Ich erkläre mein Einverständnis zur Teilnahme an der Studie und zur Speicherung meiner Antworten. Mit der Datenschutzerklärung bin ich einverstanden.
   - Ja *(PROG: 4.)*
   - Nein *(PROG: 2.)*
2. Vielen Dank, dass Sie Interesse an unserer Befragung gezeigt haben. Ohne Ihre Einwilligung in die Teilnahme- und Datenschutzerklärung ist eine Teilnahme leider nicht möglich. Sie können Ihre Auswahl noch einmal ändern oder die Befragung hier beenden
   - Ich möchte meine Zustimmung ändern *(PROG: 3.)*
   - Ich möchte die Befragung nun beenden. *(PROG: ENDE)*
3. Ich erkläre mein Einverständnis zur Teilnahme an der Studie und zur Speicherung meiner Antworten. Mit der Datenschutzerklärung bin ich einverstanden

- Ja *(PROG: 4.)*

### Inanspruchnahme von Gesundheitsleistungen

### Bitte denken Sie an Ihren eigenen Arztbesuch und Ihr eigenes Gesundheitsverhalten

1. Sind Sie in der gesetzlichen oder in der privaten Krankenversicherung krankenversichert?

- Gesetzlich versichert
- Privat versichert

1. Bei welchen Ärzten waren Sie in den letzten drei Monaten in Behandlung?

- Kein Arztbesuch
- Hausarzt
- Internist (z.B. Kardiologe, Gastroenterologe, …)
- Onkologe
- Orthopäde
- Urologe
- Gynäkologe
- Radiologe
- Psychiater bzw. Psychotherapeut
- Hals-Nasen-Ohren-Arzt
- Dermatologe (Hautarzt)
- Neurologe
- Zahnarzt bzw. Kieferchirurg
- Augenarzt
- Andere Fachrichtung

1. Wie werden bei Ihren Arztbesuchen Entscheidungen über Behandlungen und Tests getroffen?

| Entscheidung trifft  der Arzt | Entscheidung treffen Arzt und ich gemeinsam | Entscheidung treffe  ich allein |
| --- | --- | --- |

### Wahrnehmung von Überversorgung

### Bitte machen Sie einige Angaben dazu, ob und was Sie bereits von Überversorgung gehört haben und was Sie darunter verstehen.

1. Haben Sie schon einmal etwas von medizinischer Überversorgung gehört?
   - Ja *(PROG: 8.)*
   - Nein *(PROG: 9.)*
2. Was verstehen Sie unter Überversorgung?

_______________________________

*Bitte antworten Sie in Stichpunkten.*

1. Was denken Sie, könnte mit Überversorgung gemeint sein?

_______________________________

*Bitte antworten Sie in Stichpunkten.*

### Wahrnehmung von Überversorgung

### Überversorgung wird auf verschiedene Art und Weise beschrieben.

1. Wie sehr stimmen Sie den folgenden Aussagen zu?

|  |  | Stimme ganz und gar nicht zu | Stimme  eher nicht zu | Stimme  eher zu | Stimme voll und ganz zu |
| --- | --- | --- | --- | --- | --- |
| 10.1. | Überversorgung umfasst die über das Notwendige hinausgehende Behandlung von Leiden. |  |  |  |  |
| 10.2. | Überversorgung meint das schnelle Einleiten von medizinischen Maßnahmen ohne Abwarten der Selbstheilung. |  |  |  |  |
| 10.3. | Überversorgung ist eine Behandlung, die aus medizinischen Gründen nicht unbedingt notwendig wäre. |  |  |  |  |
| 10.4. | Überversorgung bezeichnet Maßnahmen, die vor allem aus finanziellen Gründen durchgeführt werden. |  |  |  |  |

### Wahrnehmung von Überversorgung

**Im deutschen Gesundheitswesen wird Überversorgung definiert als *„eine Versorgung, die über den individuellen Bedarf des Patienten hinausgehet. Dazu gehören Leistungen ohne Nutzen für den Patienten.“***

1. Haben Sie nach dieser Definition bereits Überversorgung erlebt bzw. wahrgenommen?
   - Ja
   - Nein
2. Wie schätzen Sie die Wichtigkeit von Überversorgung ein? *Bitte wählen Sie die Antwort aus, der Sie am meisten zustimmen.*
   - Weniger Überversorgung würde unser Gesundheitssystem verbessern.
   - Es gibt andere Themen, um die man sich im Gesundheitswesen kümmern sollte/ muss.
   - Überversorgung kommt zwar vor, hat aber keinen negativen Einfluss auf die Versorgung.
   - Überversorgung kommt im deutschen Gesundheitssystem nicht oft vor.
3. In welchen Bereichen vermuten Sie Überversorgung. *Bitte markieren Sie die zwei Bereiche, in denen Sie Überversorgung am häufigsten vermuten.*
   - Früherkennungs- und Vorsorgeuntersuchungen (z.B. Krebsvorsorge)
   - Verschreibung von Medikamenten
   - Operationen
   - IGeL-Leistungen (Selbstzahlerleistungen)
   - Bildgebende Verfahren (z.B. Ultraschall, Röntgen, …)
   - Blutuntersuchungen

### Wahrnehmung von Überversorgung

1. Bitte geben Sie an, bei welcher Personengruppe Sie eher Überversorgung vermuten?

|  |  | Stimme ganz und gar nicht zu | Stimme  eher nicht zu | Stimme  eher zu | Stimme voll und ganz zu |
| --- | --- | --- | --- | --- | --- |
| 14.1. | Privat Versicherte sind häufiger von Überversorgung betroffen als gesetzlich Versicherte. |  |  |  |  |
| 14.2. | Personen mit höherem Schulabschluss sind häufiger von Überversorgung betroffen als Personen mit geringem Schulabschluss. |  |  |  |  |
| 14.3. | Personen mit höherem Einkommen sind häufiger von Überversorgung betroffen als Personen mit niedrigem Einkommen. |  |  |  |  |
| 14.4. | Jüngere Personen sind häufiger von Überversorgung betroffen als ältere Personen (z.B. Rentner). |  |  |  |  |

### Ursachen von Überversorgung

### Entscheidungen für oder gegen eine diagnostische Maßnahme bzw. eine medizinische Behandlung werden von vielen Faktoren bestimmt.

1. Wo vermuten Sie eher Ursachen für ein Zuviel an Medizin?

|  |  | Stimme ganz und gar nicht zu | Stimme  eher nicht zu | Stimme  eher zu | Stimme voll und ganz zu |
| --- | --- | --- | --- | --- | --- |
| 15.1. | Je mehr Tests ein Arzt durchführt, desto kompetenter erlebe ich ihn. |  |  |  |  |
| 15.2. | Handlungsbereitschaft und Taten stellen mich eher zufrieden als Worte und Abwarten. |  |  |  |  |
| 15.3. | Ich sehe es so: Lieber einmal etwas mehr untersuchen, als einmal etwas zu übersehen. |  |  |  |  |
| 15.4. | Gerichtsprozesse führen zu Überversorgung, da sich die Ärzte in Folge diagnostisch absichern wollen. |  |  |  |  |
| 15.5. | Die Aufklärung über Nutzen und Schaden von Tests und Behandlungen scheitert am zeitlichen Aufwand. |  |  |  |  |
| 15.6. | Wenn medizinische Geräte in Praxis und Klinik vorhanden sind, werden sie auch eingesetzt. |  |  |  |  |
| 15.7. | Wenn verschiedene Ärzte sich in der Behandlung eines Patienten nicht gut abstimmen, werden mehr Leistungen erbracht. |  |  |  |  |
| 15.8. | Die Vergütung eines Tests/einer Behandlung entscheidet auch darüber, wie häufig diese/r genutzt wird. |  |  |  |  |
| 15.9. | Stress führt dazu, dass Ärzte schneller Tests anordnen. |  |  |  |  |
| 15.10. | Auch Patienten fordern medizinische Maßnahmen ein. |  |  |  |  |
| 15.11. | Wenn Ärzte unerfahren sind, ordnen Sie mehr Tests an. |  |  |  |  |

### Folgen von Überversorgung

### Eine übermäßige, über den Nutzen des Patienten hinausgehende Behandlung hat diverse Folgen.

1. Was vermuten Sie könnten Folgen von zu viel Medizin und Überversorgung sein?

|  |  | Stimme ganz und gar nicht zu | Stimme  eher nicht zu | Stimme  eher zu | Stimme voll und ganz zu |
| --- | --- | --- | --- | --- | --- |
| 16.1. | Ich kann mir gar nicht vorstellen, dass es wirklich zu viel Medizin gibt. |  |  |  |  |
| 16.2. | Ich glaube nicht, dass Medizin Schaden verursachen kann. |  |  |  |  |
| 16.3 | Das körperliche Wohlbefinden der Patienten könnte gefährdet werden. |  |  |  |  |
| 16.4. | Das seelische Wohlbefinden der Patienten könnte gefährdet werden. |  |  |  |  |
| 16.5. | Patienten könnten gegenüber Ärzten misstrauisch werden. |  |  |  |  |
| 16.6. | Die Kosten im Gesundheitswesen könnten ansteigen, sodass die Beiträge zur Krankenkasse immer teurer werden. |  |  |  |  |
| 16.7. | Je mehr medizinische Behandlungen und Tests durchgeführt werden, desto gesünder bleiben die Menschen. |  |  |  |  |

### Lösungsansätze zur Verringerung bzw. Vermeidung von Überversorgung

1. Welche Maßnahmen könnten Ihrer Meinung nach zu einer angemessenen Versorgung beitragen?

|  |  | Stimme ganz und gar nicht zu | Stimme  eher nicht zu | Stimme  eher zu | Stimme voll und ganz zu |
| --- | --- | --- | --- | --- | --- |
| 17.1. | Ich finde, dass alternativ Heilmethoden ausgebaut werden müssen. |  |  |  |  |
| 17.2. | Die ärztliche Abrechnung muss offengelegt und stärker geprüft werden. |  |  |  |  |
| 17.3 | Patienten brauchen neutrale Informationen über Behandlungsmöglichkeiten. |  |  |  |  |
| 17.4. | Wir brauchen mehr Ärzte, die jeweils weniger Patienten behandeln. |  |  |  |  |
| 17.5. | Das Nebeneinander von gesetzlicher und privater Krankenversicherung muss abgeschafft werden. |  |  |  |  |
| 17.6. | Der Austausch zwischen verschiedenen Ärzten und Behandlern muss verbessert werden. |  |  |  |  |
| 17.7. | Wenn sich Patienten stärker an den Behandlungskosten beteiligen müssten, würden weniger Untersuchungen durchgeführt werden. |  |  |  |  |
| 17.8. | Es sollte Pflicht sein, dass Patienten bei Gesundheitsproblemen zuerst den Hausarzt aufsuchen. |  |  |  |  |

### Lösungsansätze zur Verringerung bzw. Vermeidung von Überversorgung

1. In der Öffentlichkeit beginnt man, auf verschiedene Arten Überversorgung zu thematisieren. Von welchen der folgenden Kampagnen haben Sie schon einmal gehört?
   - „Choosing Wisely“
   - „Less is more“
   - „Klug entscheiden“
   - „Smarter Medicine“
   - „Quartäre Prävention“
   - Keines der genannten
2. Bitte denken Sie nun an das Gesundheitswesen in 10 Jahren. Was glauben Sie, wird es besser oder schlechter sein als zum aktuellen Zeitpunkt?
   - System wird deutlich schlechter sein
   - System wird etwas schlechter sein
   - System wird genauso gut sein wie jetzt
   - System wird etwas besser sein
   - System wird deutlich besser sein

### Demographische Angaben

### Zum Ende der Befragung würden wir Sie bitten, noch einige Angaben zu Ihrer Person und Ihren Hintergründen zu machen.

1. Bitte geben Sie Ihr Geschlecht an
   - Weiblich
   - Männlich
   - Divers
2. Bitte kreuzen Sie an, welcher Altersgruppe Sie angehören.
   - 18 bis 24 Jahre
   - 25 bis 44 Jahre
   - 45 bis 64 Jahre
   - 65 bis 84 Jahre
   - 85 Jahre oder älter
3. Welchen höchsten beruflichen Ausbildungsabschluss haben Sie?
   - (Noch) keine berufliche Ausbildung
   - Berufliche Ausbildung
   - (Fach-) Hochschulabschluss (Bachelor, Master, Diplom, …)
4. Bitte geben Sie den Status Ihrer Erwerbstätigkeit an. *Unter Erwerbstätigkeit wird jede bezahlte Tätigkeit verstanden.*
   - Nicht erwerbstätig
   - In Ausbildung/Studium
   - Erwerbstätig (als Angestellter/Arbeiter, Beamter, inklusive Mini-Job)
   - Selbstständig tätig
   - In Rente/Pension
5. Wie viele Einwohner hat Ihr Wohnort?
   - Unter 5.000
   - 5.000-20.000
   - 20.000-100.000
   - Über 100.000

### Morbidität und Gesundheitsverhalten

### Zum Ende der Befragung würden wir Sie bitten, noch einige Angaben zu Ihrer Person und Ihren Hintergründen zu machen.

1. An welchen der folgenden Gesundheitsproblemen leiden Sie?
   - Herzprobleme
   - Bluthochdruck
   - Lungenprobleme
   - Zuckerkrankheit (Diabetes)
   - Magen-Darm-Probleme
   - Nierenprobleme
   - Leberprobleme
   - Blutarmut (Anämie)
   - Gerinnungsstörung (z.B. Thrombose, Embolie)
   - Krebs
   - Depression
   - Arthrose
   - Rückenschmerzen
   - Rheuma bzw. an Autoimmunerkrankungen
   - Allergien
   - Schilddrüsenerkrankung (Über- bzw. Unterfunktion)
   - Keine der genannten Beschwerden *(PROG: 27.)*
   - Keine Gesundheitsprobleme *(PROG: 27.)*
2. Bitte kreuzen Sie an, welche der zuvor ausgewählten Beschwerden chronisch vorliegen, wegen welchen Beschwerden Sie regelmäßig Medikamente einnehmen, wegen welchen Beschwerden Sie regelmäßig zum Arzt gehen. *Sie können pro Beschwerde mehrere Antworten ankreuzen. Bitte kreuzen Sie „Keines der genannten“ an, wenn keine der drei Aussagen zutrifft.*

|  |  | Es handelt sich um eine chronische Erkrankung. | Ich nehme deswegen regelmäßig Medikamente ein. | Ich gehe deswegen regelmäßig zum Arzt. | Keines der genannten. |
| --- | --- | --- | --- | --- | --- |
| 26.1. | Herzprobleme |  |  |  |  |
| 26.2. | Bluthochdruck |  |  |  |  |
| 26.3 | Lungenprobleme |  |  |  |  |
| 26.4. | Zuckerkrankheit (Diabetes) |  |  |  |  |
| 26.5. | Magen-Darm-Probleme |  |  |  |  |
| 26.6. | Nierenprobleme |  |  |  |  |
| 26.7. | Leberprobleme |  |  |  |  |
| 26.8. | Blutarmut (Anämie) |  |  |  |  |
| 26.9. | Gerinnungsstörung (z.B. Thrombose, Embolie) |  |  |  |  |
| 26.10. | Krebs |  |  |  |  |
| 26.11. | Depression |  |  |  |  |
| 26.12. | Arthrose |  |  |  |  |
| 26.13. | Rückenschmerzen |  |  |  |  |
| 26.14. | Rheuma bzw. an Autoimmunerkrankungen |  |  |  |  |
| 26.15. | Allergien |  |  |  |  |
| 26.16 | Schilddrüsenerkrankung (Über- bzw. Unterfunktion) |  |  |  |  |

### Ende der Befragung

Vielen Dank, dass Sie an unserer Befragung teilgenommen haben. Mit Ihrer Hilfe erhalten wir ein besseres Verständnis davon, was in der Bevölkerung unter medizinischer Überversorgung verstanden wird und wo man ansetzen kann, um das Phänomen zu verringern und zu vermeiden.

1. Falls Sie noch etwas anmerken möchten, haben Sie nun die Möglichkeit. Bitte benutzen Sie hierfür das nachfolgende Fenster.

_______________________________

*Bitte antworten Sie in Stichpunkten.*

Die Ergebnisse der Studie werden voraussichtlich im August oder September 2022 auf der Website des Allgemeinmedizinischen Instituts des Universitätsklinikums Erlangen veröffentlicht.
