# Supplementary material for: What do people know and think about medical overuse? an online questionnaire study in Germany
Source: PLoS One. 2024 Mar 7;19(3):e0299907. doi: 10.1371/journal.pone.0299907 (PMC10919641; doi:10.1371/journal.pone.0299907)
Supplement: S5 File — (DOCX) [file pone.0299907.s007.docx]

# S6 File. Health problems mentioned by study participants.

|  | Total | |  |  |  |  |  | |  | |
| --- | --- | --- | --- | --- | --- | --- | --- | --- | --- | --- |
|  |  |  |  |  |  |  |  | |  | |
|  | **n** | **(%)** |  |  |  |  |  |  | |  |
| Physician’s field of medicine (multiple choice) | | | | | | | | | |  |
| Family doctor | 289 | (71.2) |  |  |  |  |  |  | |  |
| Dentist or oral surgeon | 161 | (39.7) |  |  |  |  |  |  | |  |
| Orthopedist | 86 | (21.2) |  |  |  |  |  |  | |  |
| Gynecologist | 68 | (16.7) |  |  |  |  |  |  | |  |
| Internist (e.g. cardiologist, …) | 62 | (15.3) |  |  |  |  |  |  | |  |
| Eye physician | 62 | (15.3) |  |  |  |  |  |  | |  |
| Dermatologist | 52 | (12.8) |  |  |  |  |  |  | |  |
| Ear, nose and throat physician | 50 | (12.3) |  |  |  |  |  |  | |  |
| Urologist | 44 | (10.8) |  |  |  |  |  |  | |  |
| Psychiatrist/Psychotherapist | 35 | (8.6) |  |  |  |  |  |  | |  |
| Radiologist | 30 | (7.4) |  |  |  |  |  |  | |  |
| Neurologist | 29 | (7.1) |  |  |  |  |  |  | |  |
| Other | 27 | (6.7) |  |  |  |  |  |  | |  |
| Oncologist | 4 | (1.0) |  |  |  |  |  |  | |  |
| Number of health problems per patient (multiple choice) | | | | | | | | | |  |
| None | 63 | (15.5) |  |  |  |  |  |  | |  |
| One or two | 218 | (53.7) |  |  |  |  |  |  | |  |
| Three or more | 125 | (30.8) |  |  |  |  |  |  | |  |
|  |  |  |  |  |  |  |  |  | |  |
| Total, *Median (IQR)* | 1.0 | (1.0; 3.0) |  |  |  |  |  |  | |  |
| There of: |  |  |  |  |  |  |  |  | |  |
| Chronic diseases, *Median (IQR)* | 1.0 | (0.0; 2.0) |  |  |  |  |  |  | |  |
| Need for medicine intake, *Median (IQR)* | 1.0 | (0.0; 2.0) |  |  |  |  |  |  | |  |
| Need to visit a physician, *Median (IQR)* | 0.0 | (0.0; 1.0) |  |  |  |  |  |  | |  |
|  | **Total** | | **There of** (multiple choice) | | | | | | |  |
|  |  |  | **Chronic  disease** | | **need for medication** | | **need for regular medical treatment** | | |  |
|  | **n** | **(%)** | **n** | **n** | **n** | **n** | **n** | **n** | |  |
| Health problem (multiple choice) |  |  |  |  |  |  |  |  | |  |
| Back pain | **132** | **(32.5)** | 61 | (46.2) | 37 | (28.0) | 51 | (38.6) | |  |
| Hypertension | **130** | **(32.0)** | 69 | (53.1) | 89 | (68.5) | 55 | (42.3) | |  |
| Allergies | 84 | (20.7) | 34 | (40.5) | 25 | (29.8) | 18 | (21.4) | |  |
| None | 63 | (15.5) | / |  | / |  | / |  | |  |
| Osteoarthritis | 63 | (15.5) | **43** | **(68.3)** | 7 | (11.1) | 17 | (27.0) | |  |
| Thyroid disease | 56 | (13.8) | 31 | (55.4) | 40 | (71.4) | 30 | (53.6) | |  |
| Other than mentioned | 51 | (12.6) | / |  | / |  | / |  | |  |
| Gastrointestinal disease | 51 | (12.6) | 22 | (43.1) | 15 | (29.4) | 20 | (39.2) | |  |
| Depression | 48 | (11.8) | 23 | (47.9) | 23 | (47.9) | 24 | (50.0) | |  |
| Heart disease | 41 | (10.1) | 26 | (63.4) | 26 | (63.4) | 21 | (51.2) | |  |
| Diabetes | 36 | (8.9) | 21 | (58.3) | **28** | **(77.8)** | 21 | (58.3) | |  |
| Lung disease | 30 | (7.4) | 20 | (66.7) | 15 | (50.0) | 18 | (60.0) | |  |
| Cancer | 17 | (4.2) | 4 | (23.5) | 10 | (58.8) | **14** | **(82.4)** | |  |
| Liver Disease | 13 | (3.2) | 3 | (23.1) | 5 | (38.5) | 7 | (53.8) | |  |
| Rheumatism/Autoimmune disease | 13 | (3.2) | 7 | (53.8) | 5 | (38.5) | 8 | (61.5) | |  |
| Anaemia | 12 | (3.0) | 3 | (25.0) | 4 | (33.3) | 4 | (33.3) | |  |
| Kidney disease | 11 | (2.7) | 6 | (54.5) | 3 | (27.3) | 7 | (63.6) | |  |
| Coagulation problems (e.g. thrombosis) | 9 | (2.2) | 3 | (33.3) | 5 | (55.6) | 3 | (33.3) | |  |
