# Supplementary material for: What do people know and think about medical overuse? an online questionnaire study in Germany
Source: PLoS One. 2024 Mar 7;19(3):e0299907. doi: 10.1371/journal.pone.0299907 (PMC10919641; doi:10.1371/journal.pone.0299907)
Supplement: S6 File — (DOCX) [file pone.0299907.s008.docx]

# S7 File. Results of group analysis.

# **Group differences for gender (male vs. female), knowledge of overuse (yes vs. no)**

| **Gender** | **Female**  (n = 210) | | **Male**  (n = 195) | | **U** | **Z** | **p** | **r** |
| --- | --- | --- | --- | --- | --- | --- | --- | --- |
|  | Mdn | M_Rank_ | Mdn | M_Rank_ |  |  |  |  |
| Item 15.1 | 2.0 | 191.41 | 2.0 | 215.48 | 18,041.50 | -2.22 | **.026*** | -.11 |
| Item 15.2. | 3.0 | 198.49 | 3.0 | 207.86 | 19,527.50 | -.89 | .372 |  |
| Item 15.3 | 3.0 | 210.32 | 3.0 | 195.12 | 18,938.00 | -1.49 | .137 |  |
| Item 15.4 | 2.0 | 189.39 | 3.0 | 217.66 | 17,617.00 | -2.58 | **.010*** | -.13 |
| Item 15.5 | 3.0 | 216.75 | 3.0 | 188.19 | 17,587.00 | -2.66 | **.008*** | -.13 |
| Item 15.6 | 3.0 | 197.81 | 3.0 | 208.59 | 19,384.50 | -1.02 | .308 |  |
| Item 15.7 | 3.0 | 204.75 | 3.0 | 201.12 | 20,108.00 | -.34 | .732 |  |
| Item 15.8 | 3.0 | 203.11 | 3.0 | 202.88 | 20,451.50 | -.02 | .983 |  |
| Item 15.9 | 2.0 | 193.09 | 3.0 | 213.67 | 18,394.00 | -1.90 | .058 |  |
| Item 15.10 | 3.0 | 210.91 | 3.0 | 194.48 | 18,813.00 | -1.59 | .111 |  |
| Item 15.11 | 3.0 | 181.55 | 3.0 | 226.10 | 15,970.50 | -4.12 | **<.001*** | -.20 |
| Item 16.1 | 3.0 | 206.31 | 3.0 | 199.43 | 19,779.50 | -.63 | .532 |  |
| Item 16.2 | 3.0 | 203.76 | 3.0 | 202.18 | 20,315.50 | -.14 | .885 |  |
| Item 16.3 | 3.0 | 192.80 | 3.0 | 213.99 | 18,332.00 | -2.00 | **.045*** | -.10 |
| Item 16.4 | 3.0 | 199.52 | 3.0 | 206.75 | 19,744.00 | -.68 | .499 |  |
| Item 16.5 | 3.0 | 197.53 | 3.0 | 208.89 | 19,327.00 | -1.08 | .280 |  |
| Item 16.6 | 3.0 | 206.76 | 3.0 | 198.95 | 19,685.00 | -.73 | .464 |  |
| Item 16.7 | 2.0 | 193.81 | 2.0 | 212.90 | 18,544.50 | -1.76 | .078 |  |
| Item 17.1 | 3.0 | 218.07 | 3.0 | 186.77 | 17,310.00 | -2.88 | **.004*** | -.14 |
| Item 17.2 | 3.0 | 204.73 | 3.0 | 201.14 | 20,111.50 | -.34 | .732 |  |
| Item 17.3 | 3.0 | 208.91 | 3.0 | 196.64 | 19,234.50 | -1.18 | .238 |  |
| Item 17.4 | 3.0 | 216.64 | 3.0 | 188.31 | 17,610.50 | -2.66 | **.008*** | -.13 |
| Item 17.5 | 3.0 | 218.81 | 3.0 | 185.97 | 17,154.00 | -3.00 | **.003*** | -.15 |
| Item 17.6 | 4.0 | 213.15 | 3.0 | 192.07 | 18,343.50 | -2.03 | **.042*** | -.10 |
| Item 17.7 | 2.0 | 182.40 | 3.0 | 225.18 | 16,149.50 | -3.86 | **<.001*** | -.19 |
| Item 17.8 | 2.0 | 187.55 | 3.0 | 219.64 | 17,231.00 | -2.89 | **.004*** | -.14 |
| **Knowledge of overuse** | **No**  (n = 169) | | **Yes**  (n = 237) | | **U** | **Z** | **p** | **r** |
|  | N | Median | N | Median |  |  |  |  |
| Item 15.1 | 2.0 | 204.87 | 2.0 | 201.58 | 19,702.00 | -.30 | .765 |  |
| Item 15.2. | 3.0 | 209.86 | 3.0 | 194.58 | 18,519.50 | -1.43 | .152 |  |
| Item 15.3 | 3.0 | 211.28 | 3.0 | 192.59 | 18,182.50 | -1.80 | .072 |  |
| Item 15.4 | 2.0 | 190.72 | 3.0 | 221.43 | 16,966.50 | -2.76 | **.006*** | -.14 |
| Item 15.5 | 3.0 | 195.77 | 3.0 | 214.34 | 18,194.50 | -1.70 | .089 |  |
| Item 15.6 | 3.0 | 185.92 | 3.0 | 228.16 | 15,859.00 | -3.93 | **<.001*** | -.20 |
| Item 15.7 | 3.0 | 192.35 | 3.0 | 219.14 | 17,383.50 | -2.49 | **.013*** | -.12 |
| Item 15.8 | 3.0 | 190.33 | 3.0 | 221.97 | 16,905.00 | -2.93 | **.003*** | -.15 |
| Item 15.9 | 2.0 | 187.02 | 3.0 | 226.62 | 16,120.00 | -3.60 | **<.001*** | -.18 |
| Item 15.10 | 3.0 | 190.92 | 3.0 | 221.14 | 17,044.50 | -2.89 | **.004*** | -.14 |
| Item 15.11 | 3.0 | 189.89 | 3.0 | 222.58 | 16,801.50 | -2.98 | **.003*** | -.15 |
| Item 16.1 | 3.0 | 195.92 | 3.0 | 214.13 | 18,230.50 | -1.62 | .103 |  |
| Item 16.2 | 3.0 | 198.08 | 3.0 | 211.11 | 18,741.00 | -1.18 | .240 |  |
| Item 16.3 | 3.0 | 194.35 | 3.0 | 216.33 | 17,859.00 | -2.04 | **.041*** | -.10 |
| Item 16.4 | 3.0 | 196.93 | 3.0 | 212.71 | 18,469.50 | -1.45 | .146 |  |
| Item 16.5 | 3.0 | 197.98 | 3.0 | 211.24 | 18,718.00 | -1.24 | .213 |  |
| Item 16.6 | 3.0 | 198.60 | 3.0 | 210.37 | 18,866.00 | -1.09 | .277 |  |
| Item 16.7 | 2.0 | 207.47 | 2.0 | 197.93 | 19,086.00 | -.87 | .386 |  |
| Item 17.1 | 3.0 | 201.95 | 3.0 | 205.67 | 19,659.00 | -.34 | .735 |  |
| Item 17.2 | 3.0 | 189.09 | 4.0 | 223.71 | 16,610.50 | -3.25 | **.001*** | .16 |
| Item 17.3 | 3.0 | 197.96 | 4.0 | 211.27 | 18,714.00 | -1.26 | .208 |  |
| Item 17.4 | 3.0 | 204.62 | 3.0 | 201.93 | 19,761.50 | -.25 | .804 |  |
| Item 17.5^1^ | 3.0 | 199.65 | 3.0 | 208.91 | 19,113.00 | -.84 | .404 |  |
| Item 17.6 | 3.0 | 194.51 | 4.0 | 216.10 | 17,896.50 | -2.05 | **.040*** | .10 |
| Item 17.7 | 3.0 | 190.98 | 3.0 | 221.06 | 17,059.00 | -2.67 | **.008*** | .13 |
| Item 17.8 | 3.0 | 210.23 | 2.0 | 194.07 | 18,432.00 | -1.43 | .152 |  |
