# Supplementary material for: What do people know and think about medical overuse? an online questionnaire study in Germany
Source: PLoS One. 2024 Mar 7;19(3):e0299907. doi: 10.1371/journal.pone.0299907 (PMC10919641; doi:10.1371/journal.pone.0299907)
Supplement: S8 File — (PDF) [file pone.0299907.s010.pdf]

Variable	Questionnaire Item	Description	Values	Type	Rules
record_id		REDCap ID			
redcap_survey_identifier					
studie_zu_berversorgung_fb_timestamp		timestamp of survey			
agreement	1	I declare my consent to participate in the study and to the storage of my answers. I agree with the privacy policy.	1: Yes<br>0: No	Singe Choice	If '0', jump to end_1
end_1	2	Thank you for showing interest in our survey. Without your consent to the participation and privacy policy, participation is unfortunately not possible. You can change your selection again or end the survey here.	1: I would like to change my consent<br>0: I would now like to end the questioning.	Singe Choice	If '1', jump to agreement_2<br>If '0', jump to end_2a
agreement_2	3	I give my consent to participate in the study and to the storage of my answers. I agree with the privacy policy.	1: Yes	Singe Choice	If '1', jump to insurance
insurance	4	Are you insured under the statutory or private health insurance scheme?	1: Private<br>0: Statutory	Singe Choice	
	5	**Which physicians have you seen in the last three months?**		Multiple choice	
doctor_0	5	No visit to the physician	1: Yes<br>0: No		Default: '0'<br>If '1', clear doctor_1 to doctor_14
doctor_1	5	Family doctor	1: Yes<br>0: No		Default: '0'
doctor_2	5	Internist (e.g. cardiologist, gastroenterologist, ...)	1: Yes<br>0: No		Default: '0'
doctor_3	5	Oncologist	1: Yes<br>0: No		Default: '0'
doctor_4	5	Orthopedist	1: Yes<br>0: No		Default: '0'
doctor_5	5	Urologist	1: Yes<br>0: No		Default: '0'
doctor_6	5	Gynecologist	1: Yes<br>0: No		Default: '0'
doctor_7	5	Radiologist	1: Yes<br>0: No		Default: '0'
doctor_8	5	Psychiatrist or psychotherapist	1: Yes<br>0: No		Default: '0'
doctor_9	5	Ear, nose and throat specialist	1: Yes<br>0: No		Default: '0'
doctor_10	5	Dermatologist	1: Yes<br>0: No		Default: '0'
doctor_11	5	Neurologist	1: Yes<br>0: No		Default: '0'
doctor_12	5	Dentist or oral surgeon	1: Yes<br>0: No		Default: '0'
doctor_13	5	Eye specialist	1: Yes<br>0: No		Default: '0'
doctor_14	5	Other specialty	1: Yes<br>0: No		Default: '0'
doctor_patient	6	How are decisions about treatment and tests made during your appointments with your physician?	0: Decision is made by the physician<br>100: Decision is made only by myself	Slider	
overuse_1	7	Have you ever heard of medical overuse?	1: Yes<br>0: No	Singe Choice	If '1', jump to overuse_2a<br>If '0', jump to overuse_2b
overuse_2a	8	What do you think overuse means?		Free text field	
overuse_2b	9	What do you think could be meant with overuse?		Free text field	
	10	**How much do you agree with the following statements?**			
overuse_4a	10	Overuse involves treating conditions beyond what is necessary.	1: Totally disagree<br>2: Rather disagree<br>3: Rather agree<br>4: Totally agree	Singe Choice	
overuse_4b	10	Overuse means the rapid initiation of medical procedures without waiting for self-healing.	1: Totally disagree<br>2: Rather disagree<br>3: Rather agree<br>4: Totally agree	Singe Choice	

Variable	Questionnaire Item	Description	Values	Type	Rules
overuse_4c	10	Overuse is treatment that would not be strictly necessary for medical reasons.	1: Totally disagree<br>2: Rather disagree<br>3: Rather agree<br>4: Totally agree	Singe Choice	
overuse_4d	10	Overuse refers to procedures that are carried out primarily for financial reasons.	1: Totally disagree<br>2: Rather disagree<br>3: Rather agree<br>4: Totally agree	Singe Choice	
		**In the German health care system, overuse is defined as "care that exceeds the individual needs of the patient. This includes services without benefit for the patient".**			
overuse_5	11	Have you already experienced or perceived overuse according to this definition?	1: Yes<br>0: No	Singe Choice	
overuse_6	12	How would you rate the importance of overuse? Please select the answer you agree with the most.	0: Medical overuse does not exist in our health system.<br>1: Medical overuse exists but it does not have a negative impact on healthcare provision.<br>2: There are other issues that need to be addressed.<br>3: Less medical overuse would improve our health system.	Singe Choice	
	13	**In which medical services do you suspect overuse? Please mark the two areas in which you most frequently suspect overuse.**		Multiple choice	Only allow two selections in overuse_7_0 to overuse_7_5
overuse_7_0	13	Early detection and screening (e.g. cancer screening)	1: Yes<br>0: No		Default: '0'
overuse_7_1	13	Prescription of medicine	1: Yes<br>0: No		Default: '0'
overuse_7_2	13	Surgeries	1: Yes<br>0: No		Default: '0'
overuse_7_3	13	Individual health services (IGeL)	1: Yes<br>0: No		Default: '0'
overuse_7_4	13	Imaging procedures (e.g. ultrasound, X-ray, …)	1: Yes<br>0: No		Default: '0'
overuse_7_5	13	Blood tests	1: Yes<br>0: No		Default: '0'
	14	**Please indicate which group of people you suspect are more likely to be affected by overuse?**			
overuse_8a	14	People with private health insurance are more often affected than those with statutory health insurance.	1: Totally disagree<br>2: Rather disagree<br>3: Rather agree<br>4: Totally agree	Singe Choice	
overuse_8b	14	People with a higher level of education are more often affected than people with a lower level of education.	1: Totally disagree<br>2: Rather disagree<br>3: Rather agree<br>4: Totally agree	Singe Choice	
overuse_8c	14	People with higher income are more often affected than people with lower income.	1: Totally disagree<br>2: Rather disagree<br>3: Rather agree<br>4: Totally agree	Singe Choice	
overuse_8d	14	Younger people are more often affected than older people (e.g. pensioners).	1: Totally disagree<br>2: Rather disagree<br>3: Rather agree<br>4: Totally agree	Singe Choice	
	15	**Where do you rather suspect causes for too much medicine?**			

Variable	Questionnaire Item	Description	Values	Type	Rules
reasons_1	15	I perceive a physician as more competent the more tests he performs.	1: Totally disagree<br>2: Rather disagree<br>3: Rather agree<br>4: Totally agree	Singe Choice	
reasons_2	15	Willingness to act and actions are more likely to satisfy me than words and waiting.	1: Totally disagree<br>2: Rather disagree<br>3: Rather agree<br>4: Totally agree	Singe Choice	
reasons_3	15	In my opinion, it is better to exam-ine more than to miss something.	1: Totally disagree<br>2: Rather disagree<br>3: Rather agree<br>4: Totally agree	Singe Choice	
reasons_4	15	Lawsuits lead to overuse, because physicians consequently want to protect themselves diagnostically.	1: Totally disagree<br>2: Rather disagree<br>3: Rather agree<br>4: Totally agree	Singe Choice	
reasons_5	15	Clarifying the benefits and harms of tests and treatments to patients fails due to time constraints.	1: Totally disagree<br>2: Rather disagree<br>3: Rather agree<br>4: Totally agree	Singe Choice	
reasons_6	15	If medical equipment is available in practices and clinics, it is used.	1: Totally disagree<br>2: Rather disagree<br>3: Rather agree<br>4: Totally agree	Singe Choice	
reasons_7	15	When different physicians do not coordinate well in treating a patient, more services are provided.	1: Totally disagree<br>2: Rather disagree<br>3: Rather agree<br>4: Totally agree	Singe Choice	
reasons_8	15	Payment for a diagnostic meas-ure/treatment also determines how often it is used.	1: Totally disagree<br>2: Rather disagree<br>3: Rather agree<br>4: Totally agree	Singe Choice	
reasons_9	15	Being stressed leads physicians to order tests more quickly.	1: Totally disagree<br>2: Rather disagree<br>3: Rather agree<br>4: Totally agree	Singe Choice	
reasons_10	15	Patients also request medical procedures.	1: Totally disagree<br>2: Rather disagree<br>3: Rather agree<br>4: Totally agree	Singe Choice	
reasons_11	15	If physicians are inexperienced, they are more likely to order tests.	1: Totally disagree<br>2: Rather disagree<br>3: Rather agree<br>4: Totally agree	Singe Choice	
	16	**What do you suspect might be consequences of too much medicine and overuse?**			
cons_1	16	I cannot imagine at all that there is really too much medicine.	1: Totally disagree<br>2: Rather disagree<br>3: Rather agree<br>4: Totally agree	Singe Choice	
cons_2	16	I do not believe that medicine can cause harm.	1: Totally disagree<br>2: Rather disagree<br>3: Rather agree<br>4: Totally agree	Singe Choice	
cons_3	16	The physical well-being of the patients could be endangered.	1: Totally disagree<br>2: Rather disagree<br>3: Rather agree<br>4: Totally agree	Singe Choice	

Variable	Questionnaire Item	Description	Values	Type	Rules
cons_4	16	The mental well-being of patients could be endangered.	1: Totally disagree<br>2: Rather disagree<br>3: Rather agree<br>4: Totally agree	Singe Choice	
cons_5	16	Patients might become distrustful towards physicians.	1: Totally disagree<br>2: Rather disagree<br>3: Rather agree<br>4: Totally agree	Singe Choice	
cons_6	16	Health care costs could rise, making health insurance premiums more and more expensive.	1: Totally disagree<br>2: Rather disagree<br>3: Rather agree<br>4: Totally agree	Singe Choice	
cons_7	16	The more medical treatments and tests are performed, the healthier people stay.	1: Totally disagree<br>2: Rather disagree<br>3: Rather agree<br>4: Totally agree	Singe Choice	
	17	**What measures do you think could contribute to adequate appropriate care?**			
solution_1	17	I think alternative healing methods need to be expanded.	1: Totally disagree<br>2: Rather disagree<br>3: Rather agree<br>4: Totally agree	Singe Choice	
solution_2	17	Medical billing must be disclosed and audited more closely.	1: Totally disagree<br>2: Rather disagree<br>3: Rather agree<br>4: Totally agree	Singe Choice	
solution_3	17	Patients need neutral information about treatment options.	1: Totally disagree<br>2: Rather disagree<br>3: Rather agree<br>4: Totally agree	Singe Choice	
solution_4	17	We need more physicians who each treat a smaller number of patients.	1: Totally disagree<br>2: Rather disagree<br>3: Rather agree<br>4: Totally agree	Singe Choice	
solution_5	17	The coexistence of statutory and private health insurance must be abolished.	1: Totally disagree<br>2: Rather disagree<br>3: Rather agree<br>4: Totally agree	Singe Choice	
solution_6	17	The exchange between different physicians and treatment providers must be improved.	1: Totally disagree<br>2: Rather disagree<br>3: Rather agree<br>4: Totally agree	Singe Choice	
solution_7	17	If patients had to contribute more to the cost of treatment, fewer examinations would be performed.	1: Totally disagree<br>2: Rather disagree<br>3: Rather agree<br>4: Totally agree	Singe Choice	
solution_8	17	It should be mandatory for patients to see their family physician first when they have health problems.	1: Totally disagree<br>2: Rather disagree<br>3: Rather agree<br>4: Totally agree	Singe Choice	
	18	**The public is beginning to address overuse in a variety of ways. Which of the following cam-paigns have you heard of?**		Multiple choice	
solution_11_0	18	Choosing Wisely	1: Yes<br>0: No		Default: '0'
solution_11_1	18	Less is more	1: Yes<br>0: No		Default: '0'
solution_11_2	18	Klug entscheiden	1: Yes<br>0: No		Default: '0'

Variable	Questionnaire Item	Description	Values	Type	Rules
solution_11_3	18	Smarter Medicine	1: Yes<br>0: No		Default: '0'
solution_11_4	18	Quartäre Prävention	1: Yes<br>0: No		Default: '0'
solution_11_5	18	None of the above	1: Yes<br>0: No		Default: '0'
health_system	19	Please think about the health care system in 10 years. Do you think it will be better or worse than it is now?	0: System will be significantly worse<br>1: System will be slightly worse<br>2: System will be just as good as now<br>3: System will be slightly better<br>4: System will be significantly better	Singe Choice	
gender	20	Please enter your gender	0: Female<br>1: Male<br>2: Diverse	Singe Choice	
age_group	21	Please indicate which age group you belong to.	0: 18 to 24 years<br>1: 25 to 44 years<br>2: 45 to 64 years<br>3: 65 years or older	Singe Choice	
qualification	22	What is your highest professional qualification?	0: No professional training (yet)<br>1: Professional training<br>2: (Technical) university degree (Bachelor, Master, Diploma, ...)	Singe Choice	
employment	23	Please indicate the status of your employment. Employment is understood to mean any paid activity.	0: Not employed<br>1: In professional training/student<br>2: Employed (as employee/worker, civil servant, including mini-job)<br>3: Self-employed<br>4: Retired	Singe Choice	
residence	24	How many inhabitants does your place of residence have?	0: Under 5,000<br>1: 5,000-20,000<br>2: 20,000-100,000<br>3: Over 100,000	Singe Choice	
	25	**Which of the following health problems do you suffer from?**		Multiple choice	
morbidity_1		Heart problems	1: Yes<br>0: No		Default: '0'<br>If '1', open morbdity_1_0 to morbidity_1_3
morbidity_2		Hypertension	1: Yes<br>0: No		Default: '0'<br>If '1', open morbdity_2_0 to morbidity_2_3
morbidity_3		Lung problems	1: Yes<br>0: No		Default: '0'<br>If '1', open morbdity_3_0 to morbidity_3_3
morbidity_4		Diabetes	1: Yes<br>0: No		Default: '0'<br>If '1', open morbdity_4_0 to morbidity_4_3
morbidity_5		Gastrointestinal problems	1: Yes<br>0: No		Default: '0'<br>If '1', open morbdity_5_0 to morbidity_5_3
morbidity_6		Kidney problems	1: Yes<br>0: No		Default: '0'<br>If '1', open morbdity_6_0 to morbidity_6_3
morbidity_7		Liver problems	1: Yes<br>0: No		Default: '0'<br>If '1', open morbdity_7_0 to morbidity_7_3
morbidity_8		Anemia	1: Yes<br>0: No		Default: '0'<br>If '1', open morbdity_8_0 to morbidity_8_3
morbidity_9		Coagulation disorder (e.g. thrombisis, embolism)	1: Yes<br>0: No		Default: '0'<br>If '1', open morbdity_9_0 to morbidity_9_3
morbidity_10		Cancer	1: Yes<br>0: No		Default: '0'<br>If '1', open morbdity_10_0 to morbidity_10_3
morbidity_11		Depression	1: Yes<br>0: No		Default: '0'<br>If '1', open morbdity_11_0 to morbidity_11_3
morbidity_12		Arthrosis	1: Yes<br>0: No		Default: '0'<br>If '1', open morbdity_12_0 to morbidity_12_3
morbidity_13		Back pain	1: Yes<br>0: No		Default: '0'<br>If '1', open morbdity_13_0 to morbidity_13_3

Variable	Questionnaire Item	Description	Values	Type	Rules
morbidity_14		Rheumatism or autoimmune disorder	1: Yes<br>0: No		Default: '0'<br>If '1', open morbdity_14_0 to morbidity_14_3
morbidity_15		Allergies	1: Yes<br>0: No		Default: '0'<br>If '1', open morbdity_15_0 to morbidity_15_3
morbidity_16		Thyroid disease (hyper- or hypothyroidism)	1: Yes<br>0: No		Default: '0'<br>If '1', open morbdity_16_0 to morbidity_16_3
morbidity_17		None of the above	1: Yes<br>0: No		Default: '0'<br>If '1', clear morbidity_1 to morbidity_16 and jump to end_2a
morbidity_18		No health problems	1: Yes<br>0: No		Default: '0'<br>If '1', clear morbidity_1 to morbidity_16 and jump to end_2a
	26	**Please tick which of the previously selected complaints are chronic, for which complaints you regularly take medication and for which complaints you regularly go to the physician. You can tick more than one answer per complaint. Please tick "None of the above" if none of the three statements apply.**			
morbidity_1_1	26	Heart problems: chronic disease	1: Yes<br>0: No		Default: '0'<br>Only show when morbidity_1 = 1
morbidity_1_2	26	Heart problems: regular medication intake	1: Yes<br>0: No		Default: '0'<br>Only show when morbidity_1 = 1
morbidity_1_3	26	Heart problems: regular physician visits	1: Yes<br>0: No		Default: '0'<br>Only show when morbidity_1 = 1
morbidity_1_0	26	Heart problems: none of the above	1: Yes<br>0: No		Default: '0'<br>Only show when morbidity_1 = 1<br>If '1', clear morbidity_1_1 to morbidity_1_3
morbidity_2_1	26	Hypertension: chronic disease	1: Yes<br>0: No		Default: '0'<br>Only show when morbidity_2 = 1
morbidity_2_2	26	Hypertension: regular medication intake	1: Yes<br>0: No		Default: '0'<br>Only show when morbidity_2 = 1
morbidity_2_3	26	Hypertension: regular physician visits	1: Yes<br>0: No		Default: '0'<br>Only show when morbidity_2 = 1
morbidity_2_0	26	Hypertension: none of the above	1: Yes<br>0: No		Default: '0'<br>Only show when morbidity_2 = 1<br>If '1', clear morbidity_2_1 to morbidity_2_3
morbidity_3_1	26	Lung problems: chronic disease	1: Yes<br>0: No		Default: '0'<br>Only show when morbidity_3 = 1
morbidity_3_2	26	Lung problems: regular medication intake	1: Yes<br>0: No		Default: '0'<br>Only show when morbidity_3 = 1
morbidity_3_3	26	Lung problems: regular physician visits	1: Yes<br>0: No		Default: '0'<br>Only show when morbidity_3 = 1
morbidity_3_0	26	Lung problems: none of the above	1: Yes<br>0: No		Default: '0'<br>Only show when morbidity_3 = 1<br>If '1', clear morbidity_3_1 to morbidity_3_3
morbidity_4_1	26	Diabetes: chronic disease	1: Yes<br>0: No		Default: '0'<br>Only show when morbidity_4 = 1
morbidity_4_2	26	Diabetes: regular medication intake	1: Yes<br>0: No		Default: '0'<br>Only show when morbidity_4 = 1
morbidity_4_3	26	Diabetes: regular physician visits	1: Yes<br>0: No		Default: '0'<br>Only show when morbidity_4 = 1
morbidity_4_0	26	Diabetes: none of the above	1: Yes<br>0: No		Default: '0'<br>Only show when morbidity_4 = 1<br>If '1', clear morbidity_4_1 to morbidity_4_3
morbidity_5_1	26	Gastrointestinal problems: chronic disease	1: Yes<br>0: No		Default: '0'<br>Only show when morbidity_5 = 1
morbidity_5_2	26	Gastrointestinal problems: regular medication intake	1: Yes<br>0: No		Default: '0'<br>Only show when morbidity_5 = 1
morbidity_5_3	26	Gastrointestinal problems: regular physician visits	1: Yes<br>0: No		Default: '0'<br>Only show when morbidity_5 = 1

Variable	Questionnaire Item	Description	Values	Type	Rules
morbidity_5_0	26	Gastrointestinal problems: none of the above	1: Yes<br>0: No		Default: '0'<br>Only show when morbidity_5 = 1<br>If '1', clear morbidity_5_1 to morbidity_5_3
morbidity_6_1	26	Kidney problems: chronic disease	1: Yes<br>0: No		Default: '0'<br>Only show when morbidity_6 = 1
morbidity_6_2	26	Kidney problems: regular medication intake	1: Yes<br>0: No		Default: '0'<br>Only show when morbidity_6 = 1
morbidity_6_3	26	Kidney problems: regular physician visits	1: Yes<br>0: No		Default: '0'<br>Only show when morbidity_6 = 1
morbidity_6_0	26	Kidney problems: none of the above	1: Yes<br>0: No		Default: '0'<br>Only show when morbidity_6 = 1<br>If '1', clear morbidity_6_1 to morbidity_6_3
morbidity_7_1	26	Liver problems: chronic disease	1: Yes<br>0: No		Default: '0'<br>Only show when morbidity_7 = 1
morbidity_7_2	26	Liver problems: regular medication intake	1: Yes<br>0: No		Default: '0'<br>Only show when morbidity_7 = 1
morbidity_7_3	26	Liver problems: regular physician visits	1: Yes<br>0: No		Default: '0'<br>Only show when morbidity_7 = 1
morbidity_7_0	26	Liver problems: none of the above	1: Yes<br>0: No		Default: '0'<br>Only show when morbidity_7 = 1<br>If '1', clear morbidity_7_1 to morbidity_7_3
morbidity_8_1	26	Anemia: chronic disease	1: Yes<br>0: No		Default: '0'<br>Only show when morbidity_8 = 1
morbidity_8_2	26	Anemia: regular medication intake	1: Yes<br>0: No		Default: '0'<br>Only show when morbidity_8 = 1
morbidity_8_3	26	Anemia: regular physician visits	1: Yes<br>0: No		Default: '0'<br>Only show when morbidity_8 = 1
morbidity_8_0	26	Anemia: none of the above	1: Yes<br>0: No		Default: '0'<br>Only show when morbidity_8 = 1<br>If '1', clear morbidity_8_1 to morbidity_8_3
morbidity_9_1	26	Coagulation disorder: chronic disease	1: Yes<br>0: No		Default: '0'<br>Only show when morbidity_9 = 1
morbidity_9_2	26	Coagulation disorder: regular medication intake	1: Yes<br>0: No		Default: '0'<br>Only show when morbidity_9 = 1
morbidity_9_3	26	Coagulation disorder: regular physician visits	1: Yes<br>0: No		Default: '0'<br>Only show when morbidity_9 = 1
morbidity_9_0	26	Coagulation disorder: none of the above	1: Yes<br>0: No		Default: '0'<br>Only show when morbidity_9 = 1<br>If '1', clear morbidity_9_1 to morbidity_9_3
morbidity_10_1	26	Cancer: chronic disease	1: Yes<br>0: No		Default: '0'<br>Only show when morbidity_10 = 1
morbidity_10_2	26	Cancer: regular medication intake	1: Yes<br>0: No		Default: '0'<br>Only show when morbidity_10 = 1
morbidity_10_3	26	Cancer: regular physician visits	1: Yes<br>0: No		Default: '0'<br>Only show when morbidity_10 = 1
morbidity_10_0	26	Cancer: none of the above	1: Yes<br>0: No		Default: '0'<br>Only show when morbidity_10 = 1<br>If '1', clear morbidity_10_1 to morbidity_10_3
morbidity_11_1	26	Depression: chronic disease	1: Yes<br>0: No		Default: '0'<br>Only show when morbidity_11 = 1
morbidity_11_2	26	Depression: regular medication intake	1: Yes<br>0: No		Default: '0'<br>Only show when morbidity_11 = 1
morbidity_11_3	26	Depression: regular physician visits	1: Yes<br>0: No		Default: '0'<br>Only show when morbidity_11 = 1
morbidity_11_0	26	Depression: none of the above	1: Yes<br>0: No		Default: '0'<br>Only show when morbidity_11 = 1<br>If '1', clear morbidity_11_1 to morbidity_11_3
morbidity_12_1	26	Arthrosis: chronic disease	1: Yes<br>0: No		Default: '0'<br>Only show when morbidity_12 = 1

Variable	Questionnaire Item	Description	Values	Type	Rules
morbidity_12_2	26	Arthrosis: regular medication intake	1: Yes<br>0: No		Default: '0'<br>Only show when morbidity_12 = 1
morbidity_12_3	26	Arthrosis: regular physician visits	1: Yes<br>0: No		Default: '0'<br>Only show when morbidity_12 = 1
morbidity_12_0	26	Arthrosis: none of the above	1: Yes<br>0: No		Default: '0'<br>Only show when morbidity_12 = 1<br>If '1', clear morbidity_12_1 to morbidity_12_3
morbidity_13_1	26	Back pain: chronic disease	1: Yes<br>0: No		Default: '0'<br>Only show when morbidity_13 = 1
morbidity_13_2	26	Back pain: regular medication intake	1: Yes<br>0: No		Default: '0'<br>Only show when morbidity_13 = 1
morbidity_13_3	26	Back pain: regular physician visits	1: Yes<br>0: No		Default: '0'<br>Only show when morbidity_13 = 1
morbidity_13_0	26	Back pain: none of the above	1: Yes<br>0: No		Default: '0'<br>Only show when morbidity_13 = 1<br>If '1', clear morbidity_13_1 to morbidity_13_3
morbidity_14_1	26	Rheumatism or autoimmune disorder: chronic disease	1: Yes<br>0: No		Default: '0'<br>Only show when morbidity_14 = 1
morbidity_14_2	26	Rheumatism or autoimmune disorder: regular medication intake	1: Yes<br>0: No		Default: '0'<br>Only show when morbidity_14 = 1
morbidity_14_3	26	Rheumatism or autoimmune disorder: regular physician visits	1: Yes<br>0: No		Default: '0'<br>Only show when morbidity_14 = 1
morbidity_14_0	26	Rheumatism or autoimmune disorder: none of the above	1: Yes<br>0: No		Default: '0'<br>Only show when morbidity_14 = 1<br>If '1', clear morbidity_14_1 to morbidity_14_3
morbidity_15_1	26	Allergies: chronic disease	1: Yes<br>0: No		Default: '0'<br>Only show when morbidity_15 = 1
morbidity_15_2	26	Allergies: regular medication intake	1: Yes<br>0: No		Default: '0'<br>Only show when morbidity_15 = 1
morbidity_15_3	26	Allergies: regular physician visits	1: Yes<br>0: No		Default: '0'<br>Only show when morbidity_15 = 1
morbidity_15_0	26	Allergies: none of the above	1: Yes<br>0: No		Default: '0'<br>Only show when morbidity_15 = 1<br>If '1', clear morbidity_15_1 to morbidity_15_3
morbidity_16_1	26	Thyroid disease: chronic disease	1: Yes<br>0: No		Default: '0'<br>Only show when morbidity_16 = 1
morbidity_16_2	26	Thyroid disease: regular medication intake	1: Yes<br>0: No		Default: '0'<br>Only show when morbidity_16 = 1
morbidity_16_3	26	Thyroid disease: regular physician visits	1: Yes<br>0: No		Default: '0'<br>Only show when morbidity_16 = 1
morbidity_16_0	26	Tyhroid disorder: none of the above	1: Yes<br>0: No		Default: '0'<br>Only show when morbidity_16 = 1<br>If '1', clear morbidity_16_1 to morbidity_16_3
		**Thank you for participating in our survey. With your help, we will gain a better understanding of what the population understands by medical overuse and where to start in order to reduce and avoid the phenomenon.**			
end_2a	27	If you would like to make any further comments, you now have the opportunity to do so. Please use the following window for this.		Free text field	
studie_zu_berversorgung_fb_complete		Questionnaire complete?	2: Yes		
